# Supplementary material for: Long-term HIV care outcomes under universal HIV treatment guidelines: A retrospective cohort study in 25 countries
Source: PLoS Med. 2024 Mar 18;21(3):e1004367. doi: 10.1371/journal.pmed.1004367 (PMC10962811; doi:10.1371/journal.pmed.1004367)
Supplement: S1 Text — (DOCX) [file pmed.1004367.s002.docx]

**S1 Text. Acknowledgements**

**IeDEA Asia-Pacific**

**Site investigators and study teams**

**The TREAT Asia HIV Observational Database and The TREAT Asia HIV Observational Database Low-Intensity TransfEr:** V Khol, V Ouk, C Pov, National Center for HIV/AIDS, Dermatology & STDs, Phnom Penh, Cambodia; FJ Zhang, HX Zhao, N Han, Beijing Ditan Hospital, Capital Medical University, Beijing, China; MP Lee, PCK Li, TS Kwong, TH Li, Queen Elizabeth Hospital, Hong Kong SAR, China; N Kumarasamy, C Ezhilarasi, Chennai Antiviral Research and Treatment Clinical Research Site (CART CRS), VHS-Infectious Diseases Medical Centre, VHS, Chennai, India; S Pujari, K Joshi, S Gaikwad, A Chitalikar, Institute of Infectious Diseases, Pune, India; RT Borse, V Mave, I Marbaniang, S Nimkar, BJ Government Medical College and Sassoon General Hospital, Pune, India; IKA Somia, TP Merati, AAS Sawitri, F Yuliana, Faculty of Medicine Udayana University - Prof. Dr. I.G.N.G. Ngoerah Hospital, Bali, Indonesia; E Yunihastuti, A Widhani, S Maria, TH Karjadi, Faculty of Medicine Universitas Indonesia - Dr. Cipto Mangunkusumo General Hospital, Jakarta, Indonesia; J Tanuma, S Oka, H Uemura, Y Koizumi, National Center for Global Health and Medicine, Tokyo, Japan; JY Choi, Na S, JM Kim, Division of Infectious Diseases, Department of Internal Medicine, Yonsei University College of Medicine, Seoul, South Korea; YM Gani, NB Rudi, Hospital Sungai Buloh, Sungai Buloh, Malaysia; I Azwa, A Kamarulzaman, SF Syed Omar, S Ponnampalavanar, University Malaya Medical Centre, Kuala Lumpur, Malaysia; R Ditangco, MK Pasayan, ML Mationg, Research Institute for Tropical Medicine, Muntinlupa City, Philippines; HP Chen, YJ Chan, PF Wu, Taipei Veterans General Hospital, Taipei, Taiwan; OT Ng, PL Lim, LS Lee, T Yap, Tan Tock Seng Hospital, Singapore; A Avihingsanon, S Gatechompol, P Phanuphak, C Phadungphon, HIV-NAT/Thai Red Cross AIDS Research Centre, Bangkok, Thailand; S Kiertiburanakul, A Phuphuakrat, L Chumla, N Sanmeema, Faculty of Medicine Ramathibodi Hospital, Mahidol University, Bangkok, Thailand; R Chaiwarith, T Sirisanthana, J Praparattanapan, K Nuket, Faculty of Medicine and Research Institute for Health Sciences, Chiang Mai University, Chiang Mai, Thailand; S Khusuwan, P Kambua, S Pongrapass, J Limlertchareonwanit, Chiangrai Prachanukroh Hospital, Chiang Rai, Thailand; TN Pham, KV Nguyen, DTH Nguyen, DT Nguyen, National Hospital for Tropical Diseases, Hanoi, Vietnam; CD Do, AV Ngo, LT Nguyen, Bach Mai Hospital, Hanoi, Vietnam; AH Sohn, JL Ross, B Petersen, TREAT Asia, amfAR - The Foundation for AIDS Research, Bangkok, Thailand; MG Law, A Jiamsakul, D Rupasinghe, The Kirby Institute, UNSW Sydney, NSW, Australia.

**The Australian HIV Observational Database:** *New South Wales:* D Ellis, Plaza Medical Centre, Coffs Harbour; M Bloch, D Winter, S Soma, S Macri, Holdsworth House Medical Practice, Sydney; L Burton, Lismore Sexual Health Services, Lismore; D Baker, H Farlow, E Byrne, C Williamson, East Sydney Doctors, Surry Hills; DJ Templeton, L Garton, T Doyle,  RPA Sexual Health, Camperdown; E Jackson, Nepean and Blue Mountains Sexual Health and HIV Clinic, Penrith; N Ryder, G Sweeney, B Moran, Clinic 468, HNE Sexual Health, Tamworth; A Carr, A Hawkes, K Hesse, St Vincent’s Hospital, Darlinghurst; M O’Reilly, H Farlow, R Finlayson, M Shields, R Burdon, K Muthukrishnan, R McCarthy, Taylor Square Private Clinic, Darlinghurst; K Brown, Illawarra Sexual Health Service, Warrawong; R Varma, Sydney Sexual Health Centre, Sydney; R Bopage, S Varghese, M Power, Western Sydney Sexual Health Clinic; DE Smith, Albion Street Centre; A Cogle, National Association of People living with HIV/AIDS; C Lawrence, Monash University; M Law, K Petoumenos, J Hutchinson, A Han, D Rupasinghe, WM Han, S Mokaya, The Kirby Institute, University of NSW. *Northern Territory:* M Gunathilake, S Hall, Centre for Disease Control, Darwin*. Queensland:* C Thng, C Lade, L Wang, J Lutze, S Rea, Gold Coast Sexual Health Clinic, Southport; D Russell, M Rodriguez, T Flitcroft, C Gorton, Cairns Sexual Health Service, Cairns; D Sowden, K Taing, J Broom, S Dennien, Sunshine Coast Hospital and Health Service, Nambour; D Orth, D Youds, Gladstone Road Medical Centre, Highgate Hill; A Smith, A Redmond, S Benn, J Davis, S Collins, S Smith, F Stack, Sexual Health and HIV Service in Metro North, Brisbane; *South Australia*: W Donohue, O’Brien Street General Practice, Adelaide. *Victoria:* R Moore, Northside Clinic, North Fitzroy; NJ Roth, H Lau, Prahran Market Clinic, South Yarra; R Teague, E Chow, J Ong, K Maddaford, A Levey, Melbourne Sexual Health Centre, Melbourne; J Hoy, J McMahon, M Giles, M Bryant, S Price, P Rawson-Harris, The Alfred, Melbourne; I Woolley, T Korman, J O’Bryan, K Cisera, Monash Medical Centre, Clayton. *Western Australia*: D Nolan, Department of Clinical Immunology, Royal Perth Hospital, Perth.  *New Zealand*: G Mills, Waikato District Hospital Hamilton; N Raymond, Wellington Hospital, Wellington.

**The TREAT Asia Pediatric HIV Observational Database:** V Khol, O Vichea, C Pov, National Centre for HIV/AIDS, Dermatology and STDs, Phnom Penh, Cambodia; J Tucker, New Hope for Cambodian Children, Phnom Penh, Cambodia; N Kumarasamy, E Chandrasekaran, Chennai Antiviral Research and Treatment Clinical Research Site (CART CRS), VHS-Infectious Diseases Medical Centre, VHS, Chennai, India; A Kinikar, V Mave, S Nimkar, I Marbaniang, BJ Medical College and Sassoon General Hospitals, Maharashtra, India; DK Wati, D Vedaswari, IB Ramajaya, Faculty of Medicine, Udayana University—Prof. Dr. I.G.N.G. Ngoerah Hospital, Bali, Indonesia;D Muktiarti, R Amalia, Cipto Mangunkusumo – Faculty of Medicine Universitas Indonesia, Jakarta, Indonesia; A Alam, M Lestari, D Setiabudi, R Wisaksana, S Sunarko, Hasan Sadikin General Hospital, Bandung, Indonesia; SM Fong, M Lim, F Daut, Hospital Likas, Kota Kinabalu, Malaysia; NK Nik Yusoff, P Mohamad, Hospital Raja Perempuan Zainab II, Kelantan, Malaysia; TJ Mohamed, MR Drawis, Department of Pediatrics, Women and Children Hospital Kuala Lumpur, Kuala Lumpur, Malaysia; R Nallusamy, KC Chan, Penang Hospital, Penang, Malaysia; T Sudjaritruk, V Sirisanthana, L Aurpibul, Department of Pediatrics, Faculty of Medicine, and Research Institute for Health Sciences, Chiang Mai University, Chiang Mai, Thailand; P Ounchanum, R Hansudewechakul, S Denjanta, A Kongphonoi, Chiangrai Prachanukroh Hospital, Chiang Rai, Thailand; , P Kosalaraksa, P Lumbiganon, P Tharnprisan, T Udomphanit, Division of Infectious Diseases, Department of Pediatrics, Faculty of Medicine, Khon Kaen University, Khon Kaen, Thailand; G Jourdain, PHPT-IRD UMI 174 (Institut de recherche pour le développement and Chiang Mai University), Chiang Mai, Thailand; T Puthanakit, S Anugulruengkit, W Jantarabenjakul, R Nadsasarn, Department of Pediatrics and Center of Excellence for Pediatric Infectious Diseases and Vaccines, Faculty of Medicine, Chulalongkorn University, Bangkok, Thailand; K Chokephaibulkit, K Lapphra, W Phongsamart, S Sricharoenchai, Department of Pediatrics, Faculty of Medicine Siriraj Hospital, Mahidol University, Bangkok, Thailand; QT Du, KH Truong, CH Nguyen, Children’s Hospital 1, Ho Chi Minh City, Vietnam; QN Nguyen, NM Nguyen, VC Do, VT AN, YDH Nguyen, TLT Huynh, LTT Van, Children’s Hospital 2, Ho Chi Minh City, Vietnam; LV Nguyen, DM Tran, HTT Tran, TTT Giang, National Hospital of Pediatrics, Hanoi, Vietnam; ON Le, Worldwide Orphans Foundation, Ho Chi Minh City, Vietnam; AH Sohn, JL Ross, T Suwanlerk, TREAT Asia/amfAR - The Foundation for AIDS Research, Bangkok, Thailand; MG Law, A Kariminia, The Kirby Institute, UNSW Sydney, NSW, Australia.

**IeDEA Caribbean, Central, and South America (CCASAnet)**

**Fundación Huésped, Argentina:** Pedro Cahn, Carina Cesar, Valeria Fink, Zulma Ortiz, Florencia Cahn, Agustina Roldan, Ines Aristegui, Claudia Frola.

**Instituto Nacional de Infectologia-Fiocruz, Brazil:** Beatriz Grinsztejn, Valdilea G. Veloso, Paula M. Luz, Sandra Cardoso Wagner, Ruth Friedman, Ronaldo I. Moreira, Lara Esteves Coelho, Monica Derrico Pedrosa, Guilherme Amaral Calvet, Hugo Perazzo, Rodrigo Moreira, Maria Pia Diniz Ribeiro, Mario Sergio Pereira, Emilia Moreira Jalil, Thiago Silva Torres Carolina Coutinho Mayara Secco Torres Silva

**Universidade Federal de Minas Gerais, Brazil:** Jorge Pinto, Flavia Ferreira, Marcelle Maia.

**Universidade Federal de São Paulo, Brazil:** Regina Célia de Menezes Succi, Daisy Maria Machado, Aida de Fátima Barbosa Gouvêa, Fabiana Bononi do Carmo.

**Fundación Arriarán, Chile**: Claudia Cortes, Marcelo Wolff, Maria Fernanda Rodriguez, Gabriel Castillo, Gladys Allendes.

**Les Centres GHESKIO, Haiti:** Jean William Pape, Vanessa Rouzier, Adias Marcelin, Youry Macius, Stephano Saint Preux.

**Hospital Escuela Universitario, Honduras**: Marco Tulio Luque, Diana Varela, Magda Chavez, Ada Mailhot.

**Instituto Hondureño de Seguridad Social, Honduras:** Marco Tulio Luque.

**Instituto Nacional de Ciencias Médicas y Nutrición Salvador Zubirán, Mexico**: Juan Sierra Madero, Brenda Crabtree Ramirez, Yanink Caro Vega, Alvaro López Iñiguez, Paola Alarcón Murra, Geovanna Coello, Guadalupe Muñuzuri Nájera, Lorena Guerrero Torres, Jessica Mejía, Atenea Álvarez, Sharon Ortiz

**Instituto de Medicina Tropical Alexander von Humboldt, Peru**: Eduardo Gotuzzo, Fernando Mejia, Gabriela Carriquiry.

**Vanderbilt University Medical Center, USA:** Catherine C McGowan, Stephany N Duda, Bryan E Shepherd, Timothy Sterling, Anna K Person, Peter F Rebeiro, Jessica Castilho, William C Wester, Karu Jayathilake, Fernanda Maruri, Hilary Vansell Riley, Marina Cruvinel Figueiredo, Paridhi Ranadive, Megan Turner, Gustavo Amorim, Cody Staats, Cynthia Nochowicz, Vickie Myers, Heather Burgess, Amondrea Blackman, Shengxin Tu

**Vanderbilt University, USA:** Kate Clouse, Ahra Kim

**Central Africa IeDEA**

Nimbona Pélagie, Association Nationale de Soutien aux Séropositifs et Malade du Sida (ANSS), Burundi; Patrick Gateretse, Jeanine Munezero, Valentin Nitereka, Annabelle Niyongabo, Zacharie Ndizeye , Christella Twizere, Théodore Niyongabo, Centre National de Référence en Matière de VIH/SIDA, Burundi; Hélène Bukuru, Thierry Nahimana, Martin Manirakiza,Centre de Prise en Charge Ambulatoire et Multidisciplinaire des PVVIH/SIDA du Centre Hospitalo-Universitaire de Kamenge (CPAMP-CHUK), Burundi; Patrice Barasukana, Hélène Bukuru, Martin Manirakiza, Zacharie Ndizeye, CHUK/Burundi National University, Burundi; Jérémie Biziragusenyuka, Ella Ange Kazigamwa, Centre de Prise en Charge Ambulatoire et Multidisciplinaire des PVVIH/SIDA de l’Hôpital Prince Régent Charles (CPAMP-HPRC), Burundi; Caroline Akoko, Ernestine Kesah, Esther Neba, Denis Nsame, Vera Veyieeneneng, Bamenda Regional Hospital, Cameroon; Bazil Ageh Ajeh, Rogers Ajeh, Dan Ebai Ashu, Eta Atangba, Christelle Tayomnou Deussom, Peter Vanes Ebasone, Ernestine Kendowo, Clarisse Lengouh, Gabriel Mabou, Sandra Mimou Mbunguet, Judith Nasah, Nicoline Ndiforkwah, Marc Lionel Ngamani, Eric Ngassam, George Njie Ngeke, Clenise Ngwa, Anyangwa Sidonie, Clinical Research Education and Consultancy (CRENC), Cameroon; Anastase Dzudie, CRENC and Douala General Hospital, Cameroon; Djenabou Amadou, Joseph Mendimi Nkodo, Eric Pefura Yone, Jamot Hospital, Cameroon; Annereke Nyenti, Phyllis Fon, Mercy Ndobe, Priscilia Enow, Limbe Regional Hospital, Cameroon; Catherine Akele, Akili Clever, Faustin Kitetele, Patricia Lelo, Kalembelembe Pediatric Hospital, Democratic Republic of Congo; Nana Mbonze, Guy Koba, Martine Tabala, Cherubin Ekembe, Didine Kaba, Kinshasa School of Public Health, Democratic Republic of Congo; Jean Paul Nzungani, Simon Kombela, Dany Lukeba, Sangos plus/Bomoi, Democratic Republic of Congo; Mattieu Musiku, Clement Kabambayi, Job Nsoki,Hopital de Kabinda, Democratic Republic of Congo; Merlin Diafouka, Martin Herbas Ekat, Dominique Mahambou Nsonde, CTA Brazzaville, Republic of Congo; Ursula Koukha, Adolphe Mafoua, Massamba Ndala Christ, CTA Pointe-Noire, Republic of Congo; Jules Igirimbabazi, Nicole Ayinkamiye, Bethsaida Health Center, Rwanda; Providance Uwineza, Emmanuel Ndamijimana, Busanza Health Center, Rwanda; Jean Marie Vianney Barinda, Marie Louise Nyiraneza, Gahanga Health Center, Rwanda; Marie Louise Nyiransabimana, Liliane Tuyisenge, Gikondo Health Center, Rwanda; Catherine Kankindi, Christian Shyaka, Kabuga Health Center, Rwanda; Bonheur Uwakijijwe, Marie Grace Ingabire, Kicukiro Health Center, Rwanda; Beltirde Uwamariya, Jules Ndumuhire, Masaka Health Center, Rwanda; Gerard Bunani, Fred Muyango, Nyagasambu Health Center, Rwanda; Yvette Ndoli, Oliver Uwamahoro, Nyarugunga Health Center, Rwanda; Eugenie Mukashyaka, Rosine Feza, Shyorongi Health Center, Rwanda; Chantal Benekigeri, Jacqueline Musaninyange, WE-ACTx for Hope Clinic, Rwanda; Josephine Gasana, Charles Ingabire, Jocelyne Ingabire, Faustin Kanyabwisha, Gallican Kubwimana, Fabiola Mabano, Jean Paul Mivumbi, Benjamin Muhoza, Athanase Munyaneza, Gad Murenzi, Francoise Musabyimana, Allelluia Giovanni Ndabakuranye, Fabienne Shumbusho, Patrick Tuyisenge, Francine Umwiza, Research for Development (RD Rwanda) and Rwanda Military Hospital, Rwanda; Jules Kabahizi, Janviere Mutamuliza, Boniface Nsengiyumva, Ephrem Rurangwa, Rwanda Military Hospital, Rwanda; Eric Remera, Gallican Nshogoza Rwibasira, Rwanda Biomedical Center, Rwanda. Adebola Adedimeji, Kathryn Anastos, Jean Claude Dusingize, Lynn Murchison, Viraj Patel, Jonathan Ross, Marcel Yotebieng, Natalie Zotova, Albert Einstein College of Medicine, USA; Ryan Barthel, Ellen Brazier, Heidi Jones, Elizabeth Kelvin, Denis Nash, Saba Qasmieh, Chloe Teasdale, Institute for Implementation Science in Population Health, Graduate School of Public Health and Health Policy, City University of New York (CUNY), USA; Batya Elul, Columbia University, USA; Xiatao Cai, Don Hoover, Hae-Young Kim, Chunshan Li, Qiuhu Shi, Data Solutions, USA; Kathryn Lancaster, The Ohio State University, USA; Mark Kuniholm, University at Albany, State University of New York, USA; Andrew Edmonds, Angela Parcesepe, Jess Edwards, University of North Carolina at Chapel Hill, USA; Olivia Keiser, University of Geneva; Stephany Duda; Vanderbilt University School of Medicine, USA; April Kimmel, Virginia Commonwealth University School of Medicine, USA.

**EAST AFRICA IeDEA**

Diero L, Sang E, MOI University, AMPATH Plus, Eldoret, Kenya; Bukusi E, Edwin Mulwa, George Nyanaro, KEMRI (Kenya Medical Research Institute), Kisumu, Kenya; Charles Kasozi, Mathew Ssemakadde, Masaka Regional Referral Hospital, Masaka, Uganda; Winnie Muyindike, Helen Byakwaga, Michael Kanyesigye, Mbarara University of Science and Technology (MUST), Mbarara, Uganda; Barbara Castelnuovo, Aggrey Semeere, John Michael Matovu, Infectious Diseases Institute (IDI), Mulago, Uganda; Fred Nalugoda, Francis X. Wasswa, Rakai Health Sciences Program, Kalisizo, Uganda; Paul Kazyoba, Mary Mayige, (NIMR), Dar es Salaam, Tanzania; Rita Elias Lyamuya, Francis Mayanga, Morogoro Regional Hospital, Morogoro, Tanzania; Happiness Edward Rutakulemberwa, Jerome Lwali, Tumbi Regional Hospital, Pwani, Tanzania; Denna Michael, Mark Urassa, Charles Nyaga, Richard Machemba, National Institute for Medical Research (NIMR), Kisesa HDSS, Mwanza, Tanzania; Kara Wools-Kaloustian, Constantin Yiannoutsos, Beverly Musick, Indiana University School of Medicine, Indiana University, Indianapolis, IN, USA; Batya Elul, Columbia University, New York City, NY, USA; Neelima Navuluri, Duke University, Durham, NC, USA; Rachel Vreeman, Mt. Sinai, New York, USA; Jeffrey Martin, Megan Wenger, Craig Cohen, Jayne Kulzer, University of California, San Francisco, CA, USA; Rena Patel, University of Washington, Seattle, WA, USA

**NA-ACCORD**

**NA-ACCORD Collaborating Cohorts and Representatives**:

AIDS Clinical Trials Group Longitudinal Linked Randomized Trials: Constance A. Benson and Ronald J. Bosch; AIDS Link to the IntraVenous Experience: Gregory D. Kirk; Emory-Grady HIV Clinical Cohort: Vincent Marconi and Jonathan Colasanti; Fenway Health HIV Cohort: Kenneth H. Mayer and Chris Grasso; HAART Observational Medical Evaluation and Research: Robert S. Hogg, Viviane D. Lima, Zabrina Brumme, Julio SG Montaner, Paul Sereda, Jason Trigg, and Kate Salters; HIV Outpatient Study: Kate Buchacz and Jun Li; HIV Research Network: Kelly A. Gebo and Richard D. Moore; Johns Hopkins HIV Clinical Cohort: Richard D. Moore

John T. Carey Special Immunology Unit Patient Care and Research Database, Case Western Reserve University: Jeffrey Jacobson; Kaiser Permanente Mid-Atlantic States: Michael A. Horberg; Kaiser Permanente Northern California: Michael J. Silverberg; Longitudinal Study of Ocular Complications of AIDS: Jennifer E. Thorne; MACS/WIHS Combined Cohort Study: Todd Brown, Phyllis Tien, and Gypsyamber D’Souza; Maple Leaf Medical Clinic: Graham Smith, Mona Loutfy, and Meenakshi Gupta; The McGill University Health Centre, Chronic Viral Illness Service Cohort: Marina B. Klein; Multicenter Hemophilia Cohort Study–II: Charles Rabkin; Ontario HIV Treatment Network Cohort Study: Abigail Kroch, Ann Burchell, Adrian Betts, and Joanne Lindsay; Parkland/UT Southwestern Cohort: Ank Nijhawan; Retrovirus Research Center, Universidad Central del Caribe, Bayamon Puerto Rico: Angel M. Mayor; Southern Alberta Clinic Cohort: M. John Gill; Study of the Consequences of the Protease Inhibitor Era: Jeffrey N. Martin and Steven G. Deeks; Study to Understand the Natural History of HIV/AIDS in the Era of Effective Therapy: Jun Li and John T. Brooks; University of Alabama at Birmingham 1917 Clinic Cohort: Michael S. Saag, Michael J. Mugavero, and Greer Burkholder; University of California at San Diego: Laura Bamford and Maile Karris; University of North Carolina at Chapel Hill HIV Clinic Cohort: Joseph J. Eron and Sonia Napravnik; University of Washington HIV Cohort: Mari M. Kitahata and Heidi M. Crane; Vanderbilt Comprehensive Care Clinic HIV Cohort: Timothy R. Sterling, David Haas, Peter Rebeiro, and Megan Turner; Veterans Aging Cohort Study: Lesley Park, Kathleen McGinnis, and Amy Justice

**NA-ACCORD Study Administration**:
Executive Committee: Richard D. Moore, Keri N. Althoff, Stephen J. Gange, Mari M. Kitahata, Jennifer S. Lee, Michael A. Horberg, Marina B. Klein, Rosemary G. McKaig, and Aimee M. Freeman; Administrative Core: Richard D. Moore, Keri N. Althoff, and Aimee M. Freeman; Data Management Core: Mari M. Kitahata, Stephen E. Van Rompaey, Heidi M. Crane, Liz Morton, Justin McReynolds, and William B. Lober; Epidemiology and Biostatistics Core: Stephen J. Gange, Jennifer S. Lee, Brenna Hogan, Elizabeth Humes, Sally Coburn, Wendy (Chunyan) Zheng, and Lucas Gerace
