# Supplementary material for: Long-term HIV care outcomes under universal HIV treatment guidelines: A retrospective cohort study in 25 countries
Source: PLoS Med. 2024 Mar 18;21(3):e1004367. doi: 10.1371/journal.pmed.1004367 (PMC10962811; doi:10.1371/journal.pmed.1004367)
Supplement: S1 Concept Proposal — (PDF) [file pmed.1004367.s003.pdf]

CONCEPT SHEET: MULTIREGIONAL ANALYSIS

**Date of EC approval:**	14 August 2018
**Tracking number:**	MR134
**Title:**	**Retention in care and viral load suppression among children and adults receiving HIV care under universal test and treat guidelines**
**Concept Lead:**<br>Email:	Denis Nash: denis.nash@sph.cuny.edu<br>Olga Tymejczyk: olga.tymejczyk@sph.cuny.edu<br>Ellen Brazier: ellen.brazier@sph.cuny.edu
**Collaborators:**	Matthew Law: mlaw@kirby.unsw.edu.au<br>Elizabeth Zaniewski: elizabeth.zaniewski@ispm.unibe.ch<br>Awachana Jiamsakul: ajiamsakul@kirby.unsw.edu.au<br>Azar Kariminia: akariminia@kirby.unsw.edu.au<br>Kara Wools-Kaloustian: kwools@iu.edu
**IeDEA Correspondent:**<br>Email:	Olga Tymejczyk: olga.tymejczyk@sph.cuny.edu
**Data Manager:**<br>Email:	Olga Tymejczyk: olga.tymejczyk@sph.cuny.edu
**Lead Statistician:**<br>Email:	Olga Tymejczyk: olga.tymejczyk@sph.cuny.edu
**Where will data be merged?**	City University of New York Institute for Implementation Science in Population Health (ISPH)
**Where will statistical analyses be done?**	City University of New York Institute for Implementation Science in Population Health (ISPH)
**Abstract:**<br>(±200 words)	**Background**: Recent antiretroviral treatment (ART) eligibility expansions to include all patients diagnosed with HIV eliminate an important barrier to ART uptake and subsequent viral load suppression (VLS), the goal of successful HIV treatment. We and others have shown these expansions to increase timely ART initiation following HIV care enrollment. However, even in the era of universal ART eligibility, other health system-, community-, and

patient-level barriers may constitute obstacles to retention and achievement of VLS. Monitoring patient retention on ART and the attainment of VLS among ART patients is critical for assessing progress towards UNAIDS 90-90-90 targets.

Objectives: Among ART-naïve persons newly enrolling in HIV care in countries with universal test and treat (UTT) policies:

1) Estimate retention in care (lack of death, transfer out, or loss to follow-up [LTFU]) in each 12-month interval from enrollment, as well as the proportion of retained patients with evidence of VL monitoring
2) Estimate the proportion of patients retained in care and with evidence of VLS (VL<1,000 copies/µl) in each 12-month interval from enrollment
3) Compare the proportion of patients achieving VLS at 12 months after enrollment under UTT guidelines versus before UTT adoption
4) Examine individual and site-level factors associated with VLS

Among all active patients not on ART as of the day of country-level UTT introduction:

5) Estimate the proportion of patients retained in care and with evidence of VLS (VL<1,000 copies/µl) in each 12-month interval from the date of UTT introduction.

Methods:

Data from Asia-Pacific, Central Africa, Central and South America and the Caribbean, East Africa, and North America will be used.

Descriptive statistics will be used to characterize proportions of patients with documented death and transfer out, as well as LTFU, retention, and VLS, at 12, 24, and 36 months after enrollment in HIV care. VLS at 12 months will be compared for the pre- and post-UTT periods. Multivariable logistic regression will be used to assess correlates of VLS at 12 months. Analyses will be stratified by key patient demographic, clinical, and site characteristics.

Project outline: (±1000 words)

Background

Viral load suppression (VLS) is the goal of antiretroviral treatment (ART), because of its potential to limit HIV morbidity and mortality in patients receiving treatment and to reduce onward transmission of the virus (treatment as prevention).(1-3) The importance of increasing VLS has been recognized by the Joint United Nations Programme on HIV/AIDS (UNAIDS) 90-90-90 program, which set a target of 90% VLS among patients receiving ART by 2020.(4)

ART eligibility guideline expansion is an important strategy to enable more patients to initiate ART in a timely manner and thereby potentially achieve VLS. Historically, ART eligibility expansions, which gradually extended treatment to patients in earlier stages of HIV disease, have been associated with marked increases in timely ART uptake.(5) In September 2015, the World Health Organization (WHO) recommended expansion of ART eligibility to all patients, regardless of clinical or immune status.(6) Across the world, countries have been increasingly adopting WHO's guidelines for universal testing and treatment for HIV.(7)

Although ART eligibility expansion eliminates an important barrier to accessing treatment and timely

and sustained VLS other important barriers to retention in care, adherence, and ultimately VLS persist, including health system-level factors (e.g site capacity, consistent drug supply),(8, 9) community-level factors (e.g. social support, stigma),(10-12) and patient-level factors (e.g. drug resistance,(13-16) logistical challenges, health-related beliefs)(12, 17, 18). Altogether, even in the era of universal testing and treatment, monitoring retention on ART and VLS remains of critical importance.

Primary objective

Among ART-naïve persons newly enrolling in HIV care in countries with universal test and treat (UTT) policies:

1) Estimate retention in care (lack of death, transfer out, or loss to follow-up [LTFU]) in each 12-month interval from enrollment, as well as the proportion of these patients with evidence of VL monitoring.
2) Estimate the proportion of patients retained in care and with evidence of VLS (VL<1,000 copies/µl) in each 12-month interval from enrollment.
3) Compare the proportion of patients achieving VLS at 12 months after enrollment under UTT guidelines versus before UTT adoption
4) Examine individual and site-level factors associated with VLS

Among all active patients not on ART as of the day of country-level UTT introduction:

5) Estimate the proportion of patients retained in care and with evidence of VLS (VL<1,000 copies/µl) in each 12-month interval from the date of UTT introduction.

Eligibility criteria

- Both children and adults
- Patients enrolled in HIV care after country-level introduction of UTT (depending on country, this will be 2012-2018) and in the 24 months before
- Sites with routine VL monitoring at any point since 2010 (e.g. sites which implemented routine VL monitoring in 2016 would also be eligible)

Exclusion criteria

- Sites with no pre-ART data and no VL monitoring

Primary outcomes

- Retention in care: lack of documented death, documented transfer out, or LTFU (90 days or more since last missed appointment, calculated via Haas et al. method), in each 12-month interval after enrollment
    - Retained patients will be further classified as retained on ART (if they have evidence of ART initiation) or not on ART
- VLS: VL<1,000 copies/µl, to be estimated in each 12-month period following enrollment among patients retained in HIV care. Patients receiving HIV care but not yet on ART will be assumed to have no VLS.

Other definitions

- Children: Those <10 years of age at enrollment.
- Adolescents: Those ages 10-19 at enrollment.
- Adults: Those ≥20 years at enrollment.
- Newly enrolled patients: patients with no evidence of prior HIV care, defined as any labs, visits more than 90 days before IeDEA site enrollment date.
- Patients ART-naïve at enrollment: patients with no evidence of ART exposure prior to enrollment (except for PMTCT)

- Active patients not on ART as of the day of country-level UTT introduction: patients who have 1) evidence of care (a lab, visit) within 6 months preceding UTT introduction; and 2) no evidence of ART within 12 months preceding UTT introduction. This may include transfers in to IeDEA sites and ART-experienced patients.
- ART: A regimen of at least three antiretroviral drugs.
- Routine viral load monitoring: At least one test per year.
- Country-level UTT implementation: date of national UTT policy introduction, based on a systematic ART guideline search in sources such as the UNAIDS Database of National HIV Guidelines,(19) the International Association of Providers of AIDS Care (IAPAC) Global HIV Watch,(20) health ministry websites, search engines, and published literature, including ART guideline reviews.(21-23) For countries with no publicly available ART guidelines, we obtained information from in-country HIV clinicians, researchers, and ministry of health officials.

Statistical methods

Outcomes at 12, 24, and 36 months after enrollment (among newly enrolled)

Descriptive statistics will be used to characterize the proportion of patients enrolled under UTT with documented death and transfer out of care, as well as LTFU and retention (on ART / not on ART).

Among patients retained in care at original site of enrollment, proportions with and without VLS will be calculated for each 12-month period after enrollment, with the periods defined as +/- 6 months around the dates of 12, 24, and 36 months after enrollment. In instances of multiple VLs in these time windows, the one closest to the date in question (i.e., before or after) will be used.

In sensitivity analyses, VLS at 12, 24, and 36 months since enrollment will be estimated including non-retained patients in the denominator. Using previously published ((24, 25)) and under-development (MR105) estimates, we will treat a proportion of patients LTFU or transferred out pre- and on-ART, as in care and VLS.

A parallel analysis will be conducted for sites that only provide data for ART initiators (i.e. no data are available for patients with pre-ART LTFU, death, or transfer).

Outcomes at 12, 24, and 36 months after UTT introduction (among active patients not on ART as of the day of country-level UTT introduction)

Proportion retained (on ART/not on ART) and with VLS will be calculated for 12, 24, and 36 months after UTT introduction.

VLS before vs after UTT implementation (among newly enrolled)

VLS at 12 months after enrollment will be compared among patients who enrolled 1) post-UTT, i.e. in the consecutive 6-month periods after UTT implementation; 2) in the intermediate period, 18-0 months prior to UTT implementation; and 3) pre-UTT (i.e., 24-18 months prior to UTT). The 18-month interval between the pre-UTT period and UTT adoption will allow us to apply the above-described definition of VLS at 12 months for the comparison group (using VLs in the +/- 6 month window), without crossing over into the UTT implementation period. For example, if UTT was introduced on January 1, 2015, the pre-UTT comparison group would consist of patients who enrolled in the 6-month period between January 1, 2013 and June 30, 2013, and the intermediate period - patients who enrolled between July 1, 2013 and December 31, 2014 (see schematic below).

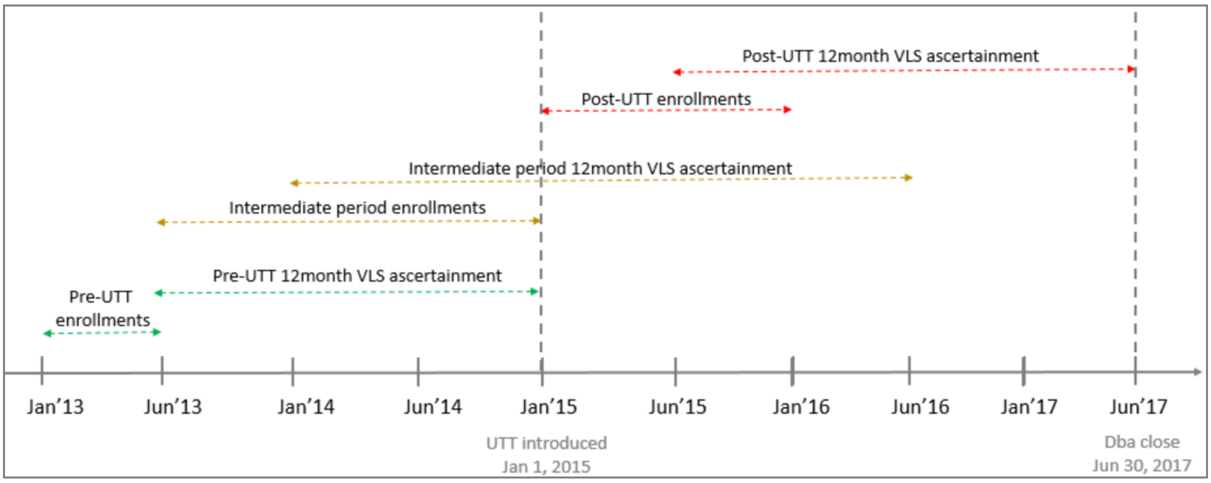

Comparable estimates for the post-UTT period only will be made for sites that introduced routine VL measurement after UTT policies were introduced in their respective countries.

A parallel analysis will be conducted for sites that only provide data for ART initiators (i.e. no data are available for patients with pre-ART LTFU, death, or transfer).

To better contextualize VLS outcomes over time, descriptive statistics will be used to characterize change in CD4 count at enrollment (% with CD4 count measured and median CD4) and initial ART regimens across the three periods.

Correlates of VLS (among newly enrolled)

Additionally, multivariable logistic regression will be completed to assess factors associated with VLS at 12 months after enrollment among patients who enrolled and initiated ART under UTT. The model will be adjusted for country income level or region, sex, age group, CD4 count at enrollment, year of enrollment, AIDS diagnosis prior to ART initiation, and initial ART regimen (NNRTI based, PI based, NNRTI/PI based, mono/dual, other).

Stratification variables

Retention analyses will be stratified by patient sex, age at enrollment, CD4 count at enrollment, period of enrollment (pre-UTT, intermediate, and post-UTT), prior AIDS diagnosis, and country income level.

VLS analyses will be stratified by the same variables, as well as class of ART drugs patients have been exposed to.

If a substantial number of patients can be linked to sites that participated in the 2017 IeDEA Site Assessment survey, estimates will also be stratified by key site characteristics such as:

- Urban/rural site location
- Facility type (level of care, private/public)

Sample size considerations

This study will include all available data from all eligible patients. No power calculations have been performed.

References

1. O'Brien WA, Hartigan PM, Martin D, Esinhart J, Hill A, Benoit S, et al. Changes in plasma

HIV-1 RNA and CD4+ lymphocyte counts and the risk of progression to AIDS. Veterans Affairs Cooperative Study Group on AIDS. N Engl J Med. 1996;334(7):426-31.
2. Joint United Nations Programme on HIV/AIDS. Public health and HIV viral load suppression. 2017.
3. Cohen MS, Chen YQ, McCauley M, Gamble T, Hosseinipour MC, Kumarasamy N, et al. Prevention of HIV-1 infection with early antiretroviral therapy. N Engl J Med. 2012;365(6):493-505.
4. Joint United Nations Programme on HIV/AIDS. 90-90-90: an ambitious treatment target to help end the AIDS epidemic. Report Geneva: UNAIDS. 2014.
5. Tymejczyk O, Brazier E, Yiannoutsos C, Wools-Kaloustian K, Althoff K, Crabtree-Ramirez B, et al. HIV treatment eligibility expansion and timely antiretroviral treatment initiation following enrollment in HIV care: A metaregression analysis of programmatic data from 22 countries. PLoS medicine. 2018;15(3):e1002534.
6. World Health Organization. Guideline on when to start antiretroviral therapy and on pre-exposure prophylaxis for HIV. Geneva, Switzerland; 2015 September 2015.
7. Ford N, Ball A, Baggaley R, Vitoria M, Low-Beer D, Penazzato M, et al. The WHO public health approach to HIV treatment and care: looking back and looking ahead. The Lancet infectious diseases. 2018;18(3):e76-e86.
8. Bain LE, Nkoke C, Noubiap JJN. UNAIDS 90-90-90 targets to end the AIDS epidemic by 2020 are not realistic: comment on "Can the UNAIDS 90-90-90 target be achieved? A systematic analysis of national HIV treatment cascades". BMJ Glob Health. 2017;2(2):e000227.
9. Levi J, Raymond A, Pozniak A, Vernazza P, Kohler P, Hill A. Can the UNAIDS 90-90-90 target be achieved? A systematic analysis of national HIV treatment cascades. BMJ Glob Health. 2016;1(2):e000010.
10. Chan BT, Tsai AC. HIV stigma trends in the general population during antiretroviral treatment expansion: analysis of 31 countries in sub-Saharan Africa, 2003-2013. Journal of acquired immune deficiency syndromes (1999). 2016;72(5):558-64.
11. Tiruneh YM, Galarraga O, Genberg B, Wilson IB. Retention in Care among HIV-Infected Adults in Ethiopia, 2005- 2011: A Mixed-Methods Study. PloS one. 2016;11(6):e0156619.
12. Topp SM, Mwamba C, Sharma A, Mukamba N, Beres LK, Geng E, et al. Rethinking retention: Mapping interactions between multiple factors that influence long-term engagement in HIV care. PloS one. 2018;13(3):e0193641.
13. Bernheimer JM, Patten G, Makeleni T, Mantangana N, Dumile N, Goemaere E, et al. Paediatric HIV treatment failure: a silent epidemic. Journal of the International AIDS Society. 2015;18:20090.
14. Cambiano V, Bertagnolio S, Jordan MR, Lundgren JD, Phillips A. Transmission of drug resistant HIV and its potential impact on mortality and treatment outcomes in resource-limited settings. The Journal of infectious diseases. 2013;207 Suppl 2:S57-62.
15. Hoffmann CJ, Maritz J, van Zyl GU. CD4 count-based failure criteria combined with viral load monitoring may trigger worse switch decisions than viral load monitoring alone. Tropical medicine & international health : TM & IH. 2016;21(2):219-23.
16. Phillips A, Shroufi A, Vojnov L, Cohn J, Roberts T, Ellman T, et al. Sustainable HIV treatment in Africa through viral-load-informed differentiated care. Nature. 2015;528(7580):S68-76.
17. Holtzman CW, Shea JA, Glanz K, Jacobs LM, Gross R, Hines J, et al. Mapping patient-identified barriers and facilitators to retention in HIV care and antiretroviral therapy adherence to Andersen's Behavioral Model. AIDS care. 2015;27(7):817-28.
18. Yehia BR, Stewart L, Momplaisir F, Mody A, Holtzman CW, Jacobs LM, et al. Barriers and facilitators to patient retention in HIV care. BMC infectious diseases. 2015;15:246.
19. Joint United Nations Programme on HIV/AIDS. UNAIDS Database of National HIV Guidelines [Available from: http://www.who.int/hiv/pub/national_guidelines/en.
20. IAPAC. Global HIV Policy Watch: International Association of Providers of AIDS Care; [Available from: http://www.hivpolicywatch.org/.
21. Gupta S, Williams B, Montaner J. Realizing the potential of treatment as prevention: global ART policy and treatment coverage. Curr HIV/AIDS Rep. 2014;11(4):479-86.

22. Gupta S, Granich R. When will sub-Saharan Africa adopt HIV treatment for all? Southern Africa Journal of HIV Medicine. 2016;17(1).
23. Ying R, Granich RM, Gupta S, Williams BG. CD4 Cell Count: Declining Value for Antiretroviral Therapy Eligibility. Clinical infectious diseases : an official publication of the Infectious Diseases Society of America. 2016;62(8):1022-8.
24. Stinson K, Ford N, Cox V, Boulle A. Patients lost to care are more likely to be viremic than patients still in care. Clinical infectious diseases : an official publication of the Infectious Diseases Society of America. 2014;58(9):1344-5.
25. Zurcher K, Mooser A, Anderegg N, Tymejczyk O, Couvillon MJ, Nash D, et al. Outcomes of HIV-positive patients lost to follow-up in African treatment programmes. Tropical medicine & international health : TM & IH. 2017;22(4):375-87.

**Ethics:**	☒ This concept uses only the IeDEA standard dataset and is covered by the core IeDEA ethics approvals.<br>☐ This concept requires additional collection of health-related data, measurements or tests, or sampling of biological material not included in the IeDEA standard dataset. Additional ethics approval is required.<br>☐ This concept does not fall into either ethics category above.<br>*Describe*:
**Dataset:**	☒ This concept requires new patient-level datasets *(likely can be combined with MR122 – concept approved, data not yet requested)*.<br>☐ This concept uses existing patient-level datasets submitted for a previous concept:<br>*Concept title:*<br>*Concept number*: MR_______________<br>☐ This concept uses IeDEA Site Assessment or other IeDEA survey data.<br>☐ This concept does not use any IeDEA data (e.g., viewpoint paper).
**Target journal(s):**	CID, JAIDS
**Milestones:**	Circulation of concept sheet: May/June 2018<br>Circulation of draft paper: January/February 2019<br>Submission to target journal: March 2019

Next Steps

Thank you for preparing a concept proposal for an IeDEA Multiregional Analysis. All IeDEA Concept Sheets are reviewed by the IeDEA Executive Committee (EC). Here are the steps for submitting your concept:

1. Before submitting the concept sheet, please **ensure all sections have been completed** or marked not applicable, the document is clean (all edits and comments are removed), and references have been added. If you are participating in an IeDEA region, ensure your Regional Principal Investigator has reviewed and approved the concept prior to submission.
2. Concepts that are developed within or have relevance to one or more IeDEA Working Groups (see list of Working Groups here) may be required to **obtain approval from the relevant IeDEA Working**

Groups before submission to the EC. Please contact Aimee Freeman (afreeman@jhu.edu) with questions on this requirement and to circulate the document to the appropriate Working Group.

3. Once the document is ready for circulation to the IeDEA Executive Committee, you can **upload it to the IeDEA Hub for EC review** at the following link:

   http://bit.ly/iedeasubmit

The concept will be reviewed by IeDEA Administrators prior to circulation to the Executive Committee. If you have questions about the form content, contact Aimee Freeman. For questions about the IeDEA Hub upload process, contact the Harmonist team at harmonist@vanderbilt.edu.
