# Supplementary material for: Long-term HIV care outcomes under universal HIV treatment guidelines: A retrospective cohort study in 25 countries
Source: PLoS Med. 2024 Mar 18;21(3):e1004367. doi: 10.1371/journal.pmed.1004367 (PMC10962811; doi:10.1371/journal.pmed.1004367)
Supplement: S1 Table — (DOCX) [file pmed.1004367.s004.docx]

**S-Table 1. Baseline characteristics among patients enrolling in care at least 24 and 36 months before database closure**

|  | **≥24 months follow-up time** | | | **≥36 months follow-up time** | | |
| --- | --- | --- | --- | --- | --- | --- |
|  | **N (%)** | **Enrolment before guideline change** | **Enrolment after guideline change** | **N (%)** | **Enrolment before guideline change** | **Enrolment after guideline change** |
| *Patient characteristics* | 64,110 | 45,098 (70.3) | 19,012 (29.7) | 54,251 | 42,859 (79.0) | 11,392 (21.0) |
| Sex |  |  |  |  |  |  |
| M | 28,694 (44.8) | 20,211 (44.8) | 8,483 (44.6) | 24,085 (44.4) | 18,841 (44.0) | 5,244 (46.0) |
| F | 35,416 (55.2) | 24,887 (55.2) | 10,529 (55.4) | 30,166 (55.6) | 24,018 (56.0) | 6,148 (54.0) |
| Age (years) |  |  |  |  |  |  |
| Median age (IQR) | 34 (27, 43) | 34 (27, 43) | 34 (27, 43) | 34 (27, 43) | 34 (27, 43) | 35 (28, 44) |
| 15-19 | 2,784 (4.3) | 1995 (4.4) | 789 (4.2) | 2,290 (4.2) | 1880 (4.4) | 410 (3.6) |
| 20-24 | 8,768 (13.7) | 6,216 (13.8) | 2,552 (13.4) | 7,189 (13.3) | 5,835 (13.6) | 1,354 (11.9) |
| 25-34 | 20,666 (32.2) | 14,478 (32.1) | 6,188 (32.5) | 17,251 (31.8) | 13,667 (31.9) | 3,584 (31.5) |
| >34 | 31,892 (49.7) | 22,409 (49.7) | 9,483 (49.9) | 27,521 (50.7) | 21,477 (50.1) | 6,044 (53.1) |
| CD4 count at enrollment |  |  |  |  |  |  |
| No CD4 count at enrollment | 37,198 (58.0) | 24,307 (53.9) | 12,891 (67.8) | 31,299 (57.7) | 23,577 (55.0) | 7,722 (67.8) |
| Any CD4 count at enrollment | 26,912 (42.0) | 20,791 (46.1) | 6,121 (32.2) | 22,952 (42.3) | 19,282 (45.0) | 3,670 (32.2) |
| Median CD4 count (IQR) | 302 (134, 495) | 299 (135, 489) | 313 (131, 519) | 297 (131, 491) | 297 (133, 489) | 295 (120, 500) |
| <200 | 9,384 (34.9) | 7,275 (35.0) | 2,109 (34.5) | 8,146 (35.5) | 6,804 (35.3) | 1,342 (36.6) |
| 200-349 | 6,023 (22.4) | 4,735 (22.8) | 1,288 (21.0) | 5,136 (22.4) | 4,359 (22.6) | 777 (21.2) |
| 350-499 | 4,962 (18.4) | 3,883 (18.7) | 1,079 (17.6) | 4,215 (18.4) | 3,581 (18.6) | 634 (17.3) |
| >=500 | 6,543 (24.3) | 4,898 (23.6) | 1,645 (26.9) | 5,455 (23.8) | 4,538 (23.5) | 917 (25.0) |
| Initiation of ART by censoring endpoint |  |  |  |  |  |  |
| Not on ART by time-point | 9,803 (15.3) | 7,774 (17.2) | 2,029 (10.7) | 7,803 (14.4) | 6,760 (15.8) | 1,043 (9.2) |
| On ART by time-point | 54,307 (84.7) | 37,324 (82.8) | 16,983 (89.3) | 46,448 (85.6) | 36,099 (84.2) | 10,349 (90.8) |
| *Clinic characteristics* |  |  |  |  |  |  |
| Location |  |  |  |  |  |  |
| Urban/mostly urban | 50,453 (78.7) | 35,384 (78.5) | 15,069 (79.3) | 43,435 (80.1) | 33617 (78.4) | 9818 (86.2) |
| Rural/mostly rural | 13,657 (21.3) | 9,714 (21.5) | 3,943 (20.7) | 10,816 (19.9) | 9242 (21.6) | 1574 (13.8) |
| Facility type |  |  |  |  |  |  |
| Health center | 15,844 (24.7) | 10,940 (24.3) | 4,904 (25.8) | 13,579 (25.0) | 10,532 (24.6) | 3,047 (26.7) |
| District hospital | 15,694 (24.5) | 10,753 (23.8) | 4,941 (26) | 12,954 (23.9) | 10,347 (24.1) | 2,607 (22.9) |
| Regional, provincial or university hospital | 28,915 (45.1) | 20,635 (45.8) | 8,280 (43.6) | 24,707 (45.5) | 19,320 (45.1) | 5,387 (47.3) |
| Unknown | 3,657 (5.7) | 2,770 (6.1) | 887 (4.7) | 3,011 (5.6) | 2,660 (6.2) | 351 (3.1) |
| Country Income level |  |  |  |  |  |  |
| Low income | 23,733 (37.0) | 16,600 (36.8) | 7,133 (37.5) | 19,512 (36.0) | 15,998 (37.3) | 3,514 (30.8) |
| Lower-middle income | 30,162 (47.0) | 20,810 (46.1) | 9,352 (49.2) | 26,288 (48.5) | 20,259 (47.3) | 6,029 (52.9) |
| Upper-middle income | 1,942 (3.0) | 1,669 (3.7) | 273 (1.4) | 1,425 (2.6) | 1,153 (2.7) | 272 (2.4) |
| High income | 8,273 (12.9) | 6,019 (13.3) | 2,254 (11.9) | 7,026 (13.0) | 5,449 (12.7) | 1,577 (13.8) |
| Year of national adoption of universal HIV treatment guidelines |  |  |  |  |  |  |
| 2012-2015 | 8,498 (13.3) | 6,088 (13.5) | 2,410 (12.7) | 7,770 (14.3) | 5,961 (13.9) | 1,809 (15.9) |
| 2016 | 49,379 (77.0) | 33,628 (74.6) | 15,751 (82.8) | 42,308 (78) | 33,087 (77.2) | 9,221 (80.9) |
| 2017-2018 | 6,233 (9.7) | 5,382 (11.9) | 851 (4.5) | 4,173 (7.7) | 3811 (8.9) | 362 (3.2) |

ART: antiretroviral therapy; IQR: interquartile range
