# Supplementary material for: Long-term HIV care outcomes under universal HIV treatment guidelines: A retrospective cohort study in 25 countries
Source: PLoS Med. 2024 Mar 18;21(3):e1004367. doi: 10.1371/journal.pmed.1004367 (PMC10962811; doi:10.1371/journal.pmed.1004367)
Supplement: S2 Table — (DOCX) [file pmed.1004367.s005.docx]

**S-Table 2: Sensitivity analyses restricted to patients enrolling 13-24 months before and 12 months after national adoption of universal HIV treatment guidelines**

| **Care outcome** | **N** | **Enrollment before guideline change***  **n (%)** | **Enrollment after guideline change**  **n (%)** | **HR** | **aHR^ǂ§^** |
| --- | --- | --- | --- | --- | --- |
| **LTC** |  |  |  |  |  |
| 12 months after enrollment | 45,095 | 5,829 (23.6) | 5,125 (25.1) | 1.09 (0.96, 1.24) | 1.05 (0.93, 1.18) |
|  | **N** | **Enrollment before guideline change***  **n (%)** | **Enrollment after guideline change**  **n (%)** | **RR** | **aRR**** |
| **Retention in care** |  |  |  |  |  |
| 12 months after ART initiation | 38,819 | 15,614 (76.0) | 12,884 (70.5) | 0.93 (0.89, 0.96) | 0.94 (0.91, 0.98) |
| **Viral load testing among patients initiating ART and retained in care** |  |  |  |  |  |
| 12 months after ART initiation | 28,498 | 9,207 (59.0) | 9,452 (73.4) | 1.24 (1.08, 1.44) | 1.24 (1.08, 1.41) |
| **Viral suppression among retained ART patients with a viral load test** |  |  |  |  |  |
| 12 months after ART initiation | 18,659 | 8,030 (87.2) | 8,163 (86.4) | 0.99 (0.96, 1.02) | 0.99 (0.97, 1.03) |

aHR: adjusted risk ratio; aRR: adjusted risk ratio; ART: antiretroviral therapy; RR: Risk ratio; HR: Risk ratio;.

* Reference group: Patients enrolling in care before adoption of universal HIV treatment guidelines.

**^ǂ^** Adjusted for sex, age group, enrollment CD4, clinic location, facility type, and country income level.

**^§^** Transfer and death treated as competing events

****** Adjusted for sex, age group, enrollment CD4, initial ART regimen type, clinic location, and country income level.
