# Supplementary material for: Long-term HIV care outcomes under universal HIV treatment guidelines: A retrospective cohort study in 25 countries
Source: PLoS Med. 2024 Mar 18;21(3):e1004367. doi: 10.1371/journal.pmed.1004367 (PMC10962811; doi:10.1371/journal.pmed.1004367)
Supplement: S3 Table — (DOCX) [file pmed.1004367.s006.docx]

**S-Table 3: Risks and hazards of LTC associated with national adoption of universal HIV treatment guidelines in countries introducing guideline changes before 2017**

| **Care outcome (N)** | **Enrollment before guideline change***  **n (%)** | **Enrollment after guideline change**  **n (%)** | **HR** | **aHR^§†^** |
| --- | --- | --- | --- | --- |
| **LTC** |  |  |  |  |
| 12 months after enrollment (59,104) | 9,316 (22.8) | 4,606 (25.2) | 1.13 (0.99, 1.28) | 1.07 (0.95, 1.20) |
| 24 months after enrollment (57,877) | 12,075 (30.4) | 6,580 (36.2) | 1.24 (1.10, 1.41) | 1.18 (1.04, 1.34) |
| 36 months after enrollment (50,078) | 14,471 (37.1) | 4,431 (40.2) | 1.13 (0.99, 1.28) | 1.14 (1.00, 1.30) |
| **LTC among patients on ART before end of follow-up** |  |  |  |  |
| 12 months after enrollment (48,083) | 5,359 (16.7) | 3,492 (21.7) | 1.37 (1.17, 1.59) | 1.3 (1.11, 1.53) |
| 24 months after enrollment (49,072) | 7,817 (23.8) | 5,296 (32.7) | 1.49 (1.29, 1.72) | 1.44 (1.24, 1.67) |
| 36 months after enrollment (42,930) | 10,089 (30.7) | 3,705 (37.0) | 1.31 (1.14, 1.51) | 1.37 (1.20, 1.56) |
| **LTC among patients not on ART before end of follow-up** |  |  |  |  |
| 12 months after enrollment (11,021) | 3,957 (45.1) | 1,114 (49.5) | 1.16 (0.99, 1.35) | 1.11 (0.93, 1.32) |
| 24 months after enrollment (8,805) | 4,258 (62.3) | 1,284 (65.1) | 1.08 (0.93, 1.25) | 1.04 (0.91, 1.19) |
| 36 months after enrollment (7,148) | 4,382 (71.4) | 726 (71.9) | 1.02 (0.89, 1.18) | 1.04 (0.92, 1.19) |

aHR: adjusted hazards ratio; ART: antiretroviral therapy; HR: Hazards ratio; LTC: Lost to clinic.

*Reference group: Patients enrolling in care before adoption of universal HIV treatment guidelines.

**^†^**Adjusted for sex, age group, enrollment CD4, facility type, clinic location, and country income level.

**^§^** Transfer and death treated as competing events
