# Supplementary material for: Long-term HIV care outcomes under universal HIV treatment guidelines: A retrospective cohort study in 25 countries
Source: PLoS Med. 2024 Mar 18;21(3):e1004367. doi: 10.1371/journal.pmed.1004367 (PMC10962811; doi:10.1371/journal.pmed.1004367)
Supplement: S4 Table — (DOCX) [file pmed.1004367.s007.docx]

**S-Table 4. Relative risks of HIV care outcomes after ART initiation associated with national adoption of universal HIV treatment guidelines in countries introducing guideline changes before 2017**

| **Care outcome (N)** | **Enrollment before guideline change***  **n (%)** | **Enrollment after guideline change**  **n (*)** | **RR** | **aRR^†^** |
| --- | --- | --- | --- | --- |
| **Retention in care** |  |  |  |  |
| 12 months after ART initiation (51,142) | 26,314 (75.9) | 11,643 (70.8) | 0.93 (0.90, 0.96) | 0.95 (0.92, 0.98) |
| 24 months after ART initiation (50,129) | 22,195 (65.7) | 9,115 (55.8) | 0.85 (0.80, 0.90) | 0.87 (0.82, 0.92) |
| 36 months after ART initiation (43,312) | 18,387 (55.3) | 4,825 (47.9) | 0.87 (0.81, 0.92) | 0.85 (0.81, 0.90) |
| **Viral load testing among patients initiating ART and retained in care** |  |  |  |  |
| 12 months after ART initiation (37,957) | 16,638 (63.2) | 8,614 (74.0) | 1.17 (1.05, 1.30) | 1.16 (1.06, 1.27) |
| 24 months after ART initiation (31,310) | 15,669 (70.6) | 6,745 (74.0) | 1.05 (0.99, 1.11) | 1.04 (0.99, 1.09) |
| 36 months after ART initiation (23,212) | 13,309 (72.4) | 3,113 (64.5) | 0.89 (0.80, 0.99) | 0.86 (0.80, 0.93) |
| **Viral suppression among those retained in care with VL testing** |  |  |  |  |
| 12 months after ART initiation (25,252) | 14,228 (85.5) | 7,382 (85.7) | 1.00 (0.98, 1.03) | 1.01 (0.98, 1.04) |
| 24 months after ART initiation (22,414) | 13,597 (86.8) | 5,990 (88.8) | 1.02 (1.01, 1.04) | 1.03 (1.01, 1.05) |
| 36 months after ART initiation (16,422) | 11,809 (88.7) | 2,818 (90.5) | 1.02 (1.01, 1.04) | 1.01 (0.99, 1.03) |

aRR: adjusted risk ratio; ART: antiretroviral therapy; RR: Risk ratio.

*Reference group: Patients enrolling in care before adoption of universal treatment guidelines.

**^†^**Adjusted for sex, age group, enrollment CD4, initial regimen type, clinic location, facility type, and country income level.
