# Supplementary material for: Long-term HIV care outcomes under universal HIV treatment guidelines: A retrospective cohort study in 25 countries
Source: PLoS Med. 2024 Mar 18;21(3):e1004367. doi: 10.1371/journal.pmed.1004367 (PMC10962811; doi:10.1371/journal.pmed.1004367)
Supplement: Attachment — Submitted filename: Response to Reviewers.docx [file pmed.1004367.s008.docx]

Louise Gaynor-Brook, MBBS PhD

Senior Editor, PLOS Medicine

Dear Dr. Gaynor-Brook,

Thank you for your consideration of our manuscript entitled “Long-term HIV care outcomes under universal HIV treatment guidelines: A retrospective cohort study in 25 countries” (PMEDICINE-D-23-02140R1) for publication in *PLOS Medicine*. We greatly appreciate the thoughtful review of our manuscript and the opportunity to revise and resubmit.

In our response to reviewer comments, we have addressed the editors’ and reviewers’ comments, noting line numbers in the clean manuscript file where these changes are reflected. We have also uploaded a version of the manuscript with changes marked. With our revision, we have also uploaded the STROBE checklist, along with a sample flow diagram and two new supplementary tables to address points raised by one of the reviewers. Additionally, in response to feedback from the editors and reviewers, in the title and throughout the manuscript, we have referenced national adoption of “universal HIV treatment” guidelines, instead of “Treat-All” guidelines to avoid potential confusion about the timing of these guideline changes, which ranged from 2012 to 2018 in the countries reflected in our analysis, as some countries adopted universal treatment guidelines prior to the World Health Organization’s “Treat All” recommendation of September 2015.

We very much appreciate the thoughtful critiques shared by the editors and reviewers and feel that addressing these comments has substantially improved our manuscript and the study. Thank you again for your consideration of this manuscript, and we look forward to hearing back from you.

Sincerely,

Ellen Brazier, PhD

**Comments from the Academic Editor:**

**Consideration of the national settings and programmes is required, which may underlie some of the results noted and may need to be addressed either by additional analysis and/or points in the Discussion.**

**The authors need to indicate and justify why there were no Southern Africa/ West Africa data included - those IeDEA data exist and most of HIV infected people live in Southern Africa. This point needs to be addressed as a limitation in the Discussion, and the text needs to be very clear of the settings from which the data come (perhaps even in the Title).**

**Wealth Index is not a sufficient covariate to indicate differences between countries. Instead there needs to be a more insightful variable that indicates something about access to health care/ access to ART/ or possibly early versus late TA policy adopters - anything that says something about the public health system would be especially important for the countries in Africa.**

**There seems to be some confusion regarding being in HIV care and being on ART - is the start of the follow up from accessing HIV care eg for testing, or from starting ART, or both? Being in HIV care may say a lot about the individual whereas ART initiation says a lot about the system with adherence then saying something about the individual too.**

**ART status is taken at censoring points but this then is likely to be strongly correlated with being in care/LTC? Instead, it would be better to allow for timing of ART initiation during the follow up period, or if not available, whether ART was already initiated at the preceding time point (whether that was a clinic visit 3 or 6 months early or the preceding censoring point).**

**People who started ART before the national TA policy was introduced are different from those starting ART after TA policy - and that is not sufficiently addressed. In particular it would be likely that those initiating before TA already have some positive link with the clinic while after TA, they may have attended a particular clinic for HIV testing (and if positive would have been initiated on ART) but then moved away and access ART elsewhere, as the initial clinic would not be the most convenient for long-term care. This needs to be addressed.**

**Before TA policies were introduced, pregnant women would already have been HIV tested and if positive would have been initiated on ART for life - this policy was common globally. The health care system would be geared towards those women and their children, even long-term. How would this have been changed by a TA policy? Can this be addressed?**

**Of course, what is most important from a public health perspective is to retain those who positively want to be on ART in long term care, in a clinic (or within a referral system that allows any move to be visible) that is convenient long term for the individual concerned. The results noted on page 17 are relevant to this point and this point should be explored in the discussion.**

We sincerely thank the Editor for considering our manuscript for publication in PLOS Medicine, and greatly appreciate the Editor’s synopsis of reviewers’ feedback above. We have addressed each of the reviewers’ comments and the editors’ requests in our revised manuscript, as discussed point-by-point below, and we believe that these changes substantially strengthen our paper. Specifically, we would like to highlight the following changes in our revised manuscript that relate to the points outlined above, with more detailed responses below.

- We agree that there may be important contextual factors influencing the comparisons we examine, which we were unable to account for. We have elaborated on this limitation in our Discussion.
- We have modified the manuscript title noting the number of countries reflected in this analysis, and we have noted as a limitation the potential non-representativeness of the clinics and countries reflected in IeDEA datasets available for this analysis. We have discussed this further, in our response to Reviewer 3.
- We strongly agree with the editor that country income levels do not capture important health system differences between countries, and that there are important differences between countries that adopted universal treatment guidelines earlier and those that adopted them later. We adjusted for clinical setting via country income level, along with health facility type and location. Additionally, we would note that the three-quarters of our sample were patients in countries where universal treatment guidelines were adopted in 2016, and relatively small proportions of our sample were from countries with earlier adoption (12.9%) or later adoption (11.7%) of such guidelines.
- Our analyses examine two groups of patients—those enrolling in HIV care and those initiating ART— by time period (before vs. after adoption of universal HIV treatment guidelines). In the former group (those enrolling in HIV care), we examine LTC at 12-month timepoints, stratified by ART status as of each timepoint. Among the latter group (those who initiate ART), we examine retention at each 12-month timepoint after ART initiation. In our revisions, we have tried to be clear about each of these groups to avoid the confusion noted by the Editor above. Additionally, in our Discussion, we have elaborated on potential differences among those who entered care and initiated ART before and after the adoption of universal treatment, and our limited ability to control for these differences.
- As the Editor rightly notes, pregnant women and children were eligible for immediate treatment prior to the adoption of universal treatment guidelines, as were people with more advanced HIV infection. While beyond the scope of the current analysis, in our conclusion, we have noted the importance of examining how universal treatment policies have affected these population groups is vitally important, particularly as Treat-All type policies may have resulted in increased “crowding out” of patients known to be at increased risk of attrition.

**Requests from the editors:**

**General comments:**

**Please include line numbers in your revised manuscript, ideally not starting from 1 with each new page.**

- We have added consecutive line numbers in the revised manuscript, as requested. All references to page numbers refer to the numbering in the unmarked (clean) version of the manuscript.

**Please use person-first language throughout e.g. “patients who are ART-naïve” rather than “ART-naïve patients”**

- Thank you for this comment. We have replaced “ART-naïve patients” with patients who are ART-naïve” as suggested and have reviewed the language throughout to use first-person language wherever appropriate.

**Throughout the paper, please adapt reference call-outs to the following style: “… every year [1,2].” (noting the absence of spaces within the square brackets).**

- We have modified the citation style as requested.

**Title: Please revise your title according to PLOS Medicine's style. We suggest “Long-term HIV care outcomes under World Health Organization “Treat-All” guidelines: A retrospective cohort study in 25 countries” or similar**

- Thank you for this suggestion. In reflecting on this point, also noted by Reviewer 3, (i.e., that some readers may be confused by the inclusion of sites from settings, like the US and Canada where universal treatment guidelines were adopted before WHO’s 2015 “Treat-All” recommendations), we have revised the title as “Long-term HIV care outcomes under universal HIV treatment guidelines: A retrospective cohort study in 25 countries,” referring to universal treatment guidelines generally, rather than WHO’s “Treat-All” guidelines. Additionally, to avoid confusion, we have used the terms “universal HIV treatment guidelines” and “guideline adoption” throughout the manuscript and tables.

**Abstract:**

**Please structure your abstract using the PLOS Medicine headings (Background, Methods and Findings, Conclusions), combining the Methods and Findings sections into one section.**

**Please note that up to 500 words are permitted.**

- Thank you for flagging this. We have restructured and expanded the abstract accordingly.

**Abstract Background: Provide expand upon the context of why the study is important. The final sentence should clearly state the study question.**

- We have expanded the Background of the abstract to include an additional sentence stating the study question (lines 25-26).

**Abstract Methods and Findings:**

**Please provide the abbreviation IeDEA**

- We have added the abbreviation, as requested.

**Please clarify what is meant by 'database closure’**

- Database closure refers to the time when clinics participating in IeDEA share their data with regional data centers which then process the data to align with IeDEA’s data exchange standards. Because of space limitations in the Abstract, we have removed reference to database closure, noting instead that our sample included 66,963 with at least 12 months of follow-up time after enrollment (line 36-67). In the methods section, however, we have clarified the term database closure for readers who are not familiar with this term (lines 160-161) and added a reference where interested readers can find more information about IeDEA’s data management and processing.

**Please provide brief demographic details of the study population (e.g. sex, age, ethnicity, etc)**

- We have summarized the age and sex of the study population (line 38-39) but have omitted ethnicity as this information is not in IeDEA’s global data.

**Please include the study design, population and settings (e.g. regions included in IeDEA), years during which data were collected, and length of follow up (eg, in mean, SD, and range).**

- We have added details related to the study design (line 27), IeDEA regions (lines 28-29), the range of years during which universal treatment guidelines were adopted in the study settings (line 31), and length of follow-up time (lines 39-40).

**Please define CI and aHR at first use.**

- We have defined these abbreviations at first use (lines 41 and 44-45).

**Please provide p values alongside 95% CIs where available.**

- In view of the debates surrounding p-values, which Reviewer 1 highlighted in their comments, we hesitate to add p-values to the abstract, particularly as 95% confidence intervals are reported for our estimates of the adjusted hazards ratios at 12, 24, and 36 months. In addition, the large sample size of our study makes p-values less helpful for those that rely on them to look for ‘significant differences’. However, if the Editors consider p-values necessary, we will add them.

**Please specify ‘months’ after 12 and 24 when discussing hazards of LTC**

- We have specified ‘months’ where noted (line 45).

**Please include the important dependent variables that are adjusted for in the analyses.**

- We have specified the variables used in confounder adjustment (lines 42-43).

**In the last sentence of the Abstract Methods and Findings section, please describe 2-3 of the main limitations of the study's methodology.**

- We have summarized the study’s main limitations, as indicated (lines 52-54).

**Abstract Conclusions:**

**Please begin your Abstract Conclusions with "In this study, we observed ..." or similar, to summarize the main findings from your study, without overstating your conclusions. Please emphasize what is new and address the implications of your study, being careful to avoid assertions of primacy.**

- We have modified the Abstract Conclusion as recommended (lines 55-58).

**Author Summary:**

**At this stage, we ask that you include a short, non-technical Author Summary of your research to make findings accessible to a wide audience that includes both scientists and non-scientists. The Author Summary should immediately follow the Abstract in your revised manuscript. This text is subject to editorial change and should be distinct from the scientific abstract. Please see our author guidelines for more information. In the final bullet point of ‘****What Do These Findings Mean?’, please describe the main limitations of the study in non-technical language.**

- We have revised our previous summary to align with PLOS Med’s guidelines (lines 60-110).

**Introduction:**

**Please expand a little on the background to your study and address other past research.**

- We have expanded the introduction section and cited all relevant research identified through our literature review (lines 123-134).

**Methods:**

**Did your study have a prospective protocol or analysis plan? Please state this (either way) early in the Methods section. If a prospective analysis plan was used in designing the study, please include the relevant prospectively written document with your revised manuscript as a Supporting Information file to be published alongside your study, and cite it in the Methods section. If no such document exists, please make sure that the Methods section transparently describes when analyses were planned, and if/when reported analyses differed from those that were planned. Changes in the analysis-- including those made in response to peer review comments-- should be identified as such in the Methods section of the paper, with rationale. If a reported analysis was performed based on an interesting but unanticipated pattern in the data, please be clear that the analysis was data-driven.**

- Thank you for this query. We noted in the Methods section that key elements of our study were based on an initial concept proposal approved by the IeDEA collaboration (lines 150-151, 244-246). We have also noted that competing risks and cause-specific hazards regression were introduced a priori to ensure that estimates of interest accounted for competing events (lines 246-248). Additionally, we now note that a second sensitivity analysis was undertaken in response to Reviewer 1’s feedback (lines 248-252).

**Please define NRTI+NNRTI at first use,**

- Thank you for noting this undefined abbreviation. We have corrected the text (line 203).

**Please provide the names of the institutional review boards that provided ethical approval.**

- We have added the name of the City University of New York’s institutional review board, as requested (lines 152-153).

**Please ensure that the study is reported according to the STROBE guideline, and include the completed STROBE checklist as Supporting Information. Please add the following statement, or similar, to the Methods: "This study is reported as per the Strengthening the Reporting of Observational Studies in Epidemiology (STROBE) guideline (S1 Checklist)." When completing the checklist, please use section and paragraph numbers, rather than page numbers which will likely no longer correspond to the appropriate sections after copy-editing.**

- We have completed the STROBE checklist and included it as S1 Checklist.

**Please report the years during which data used in this study were collected.**

- We have noted the years of data used in the Methods section (lines 166-167).

**Results:**

**Please define IQR at first use.**

- We have corrected the text (line 274).

**In the first, third and fifth sentences on page 7, please specify that the results presented apply to all patients in the cohort (i.e. not defined by before/after Treat All)**

- Thank you for highlighting these sentences. We have specified that these results are for all patients, as suggested (lines 301, 313).

**Please quantify the results presented in the main text, providing 95% CIs and exact p values.**

- P-values for descriptive statistics have been reported in the corresponding tables of the manuscript (Table 1 and 2), and we have reviewed the text to ensure that 95% confidence intervals are consistently reported for outcomes of interest. Because of current debates surrounding the usefulness of p-values, which Reviewer 1 highlighted in their feedback, and because of our study’s large sample size, we have limited the reporting of p-values to the tables themselves. However, if the Editors recommend adding p-values throughout the text, we will revise accordingly.

**Please indicate which factors are adjusted for the main text relating to Table 5.**

- Thank you for this suggestion. We have added these details to the Methods section (lines 233-234)

**Where aHR/aRR are provided, please ensure to specify the comparison group.**

- In accordance with Reviewer 1’s feedback, we have omitted the analysis examining factors associated with LTC, to which we believe this feedback applied.

**Please define the length of follow up (eg, in mean, SD, and range).**

- We have summarized the length of follow-up time as suggested (lines 268-269).

**Discussion:**

**Please present and organize the Discussion as follows: a short, clear summary of the article's findings; what the study adds to existing research and where and why the results may differ from previous research; strengths and limitations of the study; implications and next steps for research, clinical practice, and/or public policy; one-paragraph conclusion. Please remove the Conclusions subheading within your Discussion**

- Thank you for this guidance. We have revised the Discussion in accordance with the recommended structure.

**Please remove the information on competing interests, funding and data sharing from the**

**end of the main text. In the event of publication, this information will appear in the article**

**metadata, via entries in the submission form.**

- We have removed this information from the manuscript file, as requested.

**Figures:Please define all abbreviations used in the figure legend of each figure.**

- We confirm that all abbreviations used in the figures have been defined in the figure legend as outlined in PLOS Guidelines to Authors.

**Tables:Please define all abbreviations used in the table legend of each table, including in the supplementary files.**

- We confirm that all abbreviations used in each table are defined in the table legend.

**When a p value is given, please specify the statistical test used to determine it in the legend.**

**Please provide exact p values, rather than e.g.<.001**

- We have added a footnote to Table1 and Table 2 specifying the statistical test used and confirm that all p-values are exact p-values, apart from those <.0001.

**References:**

**Please ensure that journal name abbreviations match those found in the National Center for Biotechnology Information (NCBI) databases), and are appropriately formatted and capitalised. Six authors should be listed prior to ‘et al’. Please also see for further details on reference formatting.**

- Thank you for flagging this. We have corrected the citation formatting.

**Reviewer #1:**

**In this paper, the authors present an analysis of data from the IeDEA consortium, aiming to answer the question of whether people with HIV had better follow-up outcomes following the introduction of Treat-All guidelines. While this analysis does account for the competing risk of death when assessing the effect of the Treat-All guidelines on the outcome of care retention, there are several aspects of this analysis that require clarification and further justification, as described in my comments below.**

- We appreciate Reviewer 1’s thoughtful consideration of our manuscript and constructive suggestions. We have endeavored to address each point in our revised manuscript, as outlined below.

**1. Please complete and include the STROBE checklist**

- We have completed the STROBE checklist and included it as S1 Checklist.

**2. Abstract: The regions that the 25 countries were drawn from and the period of time that the data corresponds to should be mentioned.**

- We have noted the regions from which countries are drawn for this analysis (lines 28-29), as well as the years during which guideline changes occurred in these countries (line 31).

**3. Methods:**

**a. What statistical program was used to analyse the data? If R, please be sure to cite the relevant packages.**

- We have noted the statistical program used for all analyses (SAS 9.4) (line 254)

**b. It is stated that multivariable cause-specific hazards regression was applied - but what model specifically was assumed? How was clustering within clinic accounted for in the cause-specific hazards regression models? Please provide details.**

- Thank you for noting these points. We have clarified that we used Cox proportional hazards regression models (line 223). Clustering within clinic was accounted for by using the option of COVSANDWICH in the PROC PHREG statement with the ID statement used to indicate that observations within the same clinic were from the same cluster. We have not added these SAS-specific details to the manuscript, but will revise accordingly if so advised.

**c. What is meant by "correlates of LTC"? What research question is this analysis aiming to answer? Generally speaking, if interest is in the effect of a covariate or an exposure on an outcome, the researcher must consider all variables that may act as confounders for the relationship between that covariate/exposure on the outcome, and adjust for these in an analysis. Thus, separate analyses may be required for each exposure/covariate and outcome pair. Tables of regression coefficients or hazard ratios as presented in Table 4 are very difficult to interpret, and often do not have a valid causal interpretation and thus should not be included. Please see Westreich D and Greenland S, The Table 2 Fallacy: presenting and interpreting confounder and modifier coefficients. American Journal of Epidemiology, 2013;177:92-298.**

- Thank you for noting these points. We agree with the reviewer that the estimates presented in Table 4 do not have a valid causal interpretation and that separate analyses exploring the association of each covariate with the LTC outcome, controlling for hypothesized counders of that relationship would be more appropriate. While PLOS Med and other journals frequently publish similar multivariable analyses exploring factors associated with a particular outcome of interest, we have removed Table 4 from the paper, along with all mention of this analysis from the Methods, Results and Discussion sections.

**d. I would think that those patients with ART initiation are likely to have been treated for longer at a clinic than those patients without ART initiation, and thus would have a lower incidence of loss to follow up. This does not appear to have been commented on: it has implications for interpretation of results. The analysis of patients who commenced ART during their follow up is conditioning on a future event; a more appropriate analysis would treat ART initiation as a time-varying exposure. Further, after the Treat-All guidelines were introduced I can imagine that more patients commenced ART earlier; thus the effect of the introduction of the guidelines in those patients who commenced ART cannot be disentangled from the differences between the group of patients who had commenced ART before the introduction of Treat-All and the group who commenced ART after Treat-All. The approach taken requires justification or deletion.**

- We appreciate these nuanced points. These considerations were an important factor in the design of our study, and particularly our decision to look at LTC (Table 3) after the date of enrollment in HIV care (overall and by ART initiation status), as well as our decision to look at care retention at 12-month time points after ART initiation (Table 4), among those patients initiating ART. The timeframe for this latter analysis starts at the point of ART initiation. Based on the Reviewer’s comments, and those of another reviewer 3, we have added a row to Table 1, reporting the mean time from enrollment to treatment initiation before and after guideline change, and have referenced these data in our Results section (lines 280-284) and in the Discussion (lines 386-387). We have also ackowledged that lower retention among patients on ART in the era of universal HIV treatment may reflect the fact that some individuals who would have been LTC prior to ART initiation before guideline adoption initiated ART more rapidly in the universal treatment era, changing the LTC profile of ART initiators (lines 431-433).

**e. Patients who were enrolled in clinics prior to the date of adoption in their country will, depending on their date of enrolment, still be enrolled after the date of adoption. Thus patients could have a time-varying exposure to the Treat-All policy. How was this accounted for in the analysis? What are the impacts of this on conclusions?**

- Thank you for noting these points. This was indeed something we considered and it motivated the sensitivity analysis which is reported in Supplementary Table 2. As shown in Figure 2, we restricted the sample of patients enrolling before the era of universal HIV treatment to those enrolling 13-24 months before guideline change to avoid the overlap between their outcome ascertainment window and the introduction of Treat-All, and the results were consistent with our main analysis. However, as noted by the Reviewer, for the patients enrolling in care and initiating ART before the Treat All era, longer-term outcomes at 24 and 36 months occurred during the period after the adoption of expanded treatment guidelines. We have noted this in our revisions (lines 441-443).

**4. Results**

**a. How complete was the dataset? Were all patient-level characteristics available for all patients in the dataset, or was the analysis restricted to those patients with complete data? Missing CD4 count at enrolment is mentioned, but what about other characteristics? If there was missingness in other characteristics, what implications might this have for the results and their interpretation?**

- Thank you for this query. We used complete case analysis, and apart from missing data related to CD4 at enrollment, few otherwise-eligible patients excluded from the analysis for missing data related to patient sex (n=1), missing age or implausible age at care enrollment (n=28), or missing death date (n=134). Given our robust sample size, we do not think the exclusion of these patients affected the results we report. In our revision, we have elucidated the number of patients excluded from the analysis for ineligibility and for data missingness (lines 261-265), and we have added a sample flow diagram (Figure 3).

**b. In several places on Page 7, results relating to proportions of patients experiencing outcomes are mentioned without any accompanying proportions or indications of where readers may find these. For example, in the first paragraph: where are the proportions of patients with transfers at each of the time points mentioned in the second sentence?**

- Thank you for this comment. We had referenced Table 2 in the previous sentence (line 303), where the estimates are reported. However, in response to the Reviewer’s comment, we now also report these proportions in the text (lines 305-311 and line 315-316).

**c. The modern usage of p-values favours a more holistic interpretation of results, rather than simply assessing whether a p-value happens to be small. When a large amount of data is analysed, small differences can have very small p-values: statistical significance may not equate to statistical significance. The American Statistical Association's statement on p-values, provides further context and guidance.** **An assessment of the differences between groups guided by clinical rather than statistical significance, with consideration of variability, would be far more appropriate here.**

- We agree with the reviewer’s concern about reliance on p-values, particularly as differences that are not substantive will be statistically significant when sample sizes are large. We are familiar with the ASA’s statement on p-values, and had avoided references to p-values in the manuscript, focusing instead on 95% confidence intervals in what are now Tables 3 and 4, because confidence intervals are more informative about the precision of estimates. Where the differences we report are likely not substantive from a clinical perspective, we have acknowledged this (lines 362-364).

**d. As indicated in my comments on the Statistical Analysis section, it is extremely difficult to interpret the results presented in Table 4 in a useful way. Thus this table and associated discussion should be deleted.**

- As noted above, we have removed the previous Table 4 from our manuscript and all discussion of the results of this analysis.

**5. Discussion**

**a. For those countries with national adoption dates occurring in 2017-2018, the disruption caused by the COVID-19 pandemic and associated restrictions could have had a large impact on outcomes at 24 to 36 months. Although this is mentioned in the discussion, a sensitivity analysis where data from 2017 and 2018 is excluded is required.**

- Thank you for this suggestion. As shown in Table 1, the number of patients included from countries with adoption of universal HIV treatment guidelines in 2017 and 2018 (and whose 24- and 36-month outcome windows could have overlapped with the COVID-19 pandemic was relatively small (7,859 patients or 11.7%) of our sample. In response to this comment, we did additional sensitivity analyses, excluding patients from countries with later adoption of universal treatment guidelines, and the results were consistent with our main analyses reported in Table 3 and Table 4. Two additional tables detailing these sensitivity analyses have been added to the Supplementary tables.

**b. Given my concerns above regarding the analysis of the patients who had initiated ART, the conclusions regarding decreased retention after ART initiation need to be further justified.**

- We agree with the reviewer that there may be important differences between patients initiating ART and those not on ART, and as noted above, we examined LTC and retention in two groups of patients—those enrolling in HIV care and those initiating ART—by time period of enrollment (before vs. after adoption of universal HIV treatment guidelines). While LTC among the former group is complicated by variability in the time to ART initiation, we believe this concern is somewhat mitigated for the latter group but have acknowledged the concern in our Discussion (lines 421-423).

**Reviewer #2:**

**This is a large, retrospective cohort analysis by the IeDEA group, using data from 109 clinics in 25 countries to study the impact of the Treat-All (TA) policy on retention on ART, viral load (VL) coverage and VL suppression. The manuscript is very well written, the complex methodology has been explained clearly and the results are nicely displayed in graphs and tables. There are no ethical issues.**

- Thank you for your review and consideration of our manuscript. We appreciate the Reviewer’s feedback on our study and suggestions for revision.

**Major points**

**1. The main benefit of TA is higher uptake of ART and prevention of complications through earlier treatment. While uptake of ART among persons diagnosed and enrolled in care will be higher after TA, this benefit could be reduced or negated by higher attrition from care. In other words, of persons enrolled in care, the net percentage of those who started ART and were then retained on ART at the 3 time points, should be compared between the 2 time periods. Does the study provide this insight? The authors have stratified LTC between those starting ART and not starting ART, and compared retention among those who started ART, but this is not the same. Can the authors explain?**

- We agree with the reviewer that the adoption of universal HIV treatment guidelines has resulted in earlier treatment of HIV among the population of people enrolling in care, which holds promise for both individual and population health outcomes. We also agree that even if treatment uptake increases under universal treatment guidelines, this benefit could be negated if attrition is higher. This is the primary question that motivated our analysis. We sought to explore this in two ways. First, we examined LTC after enrollment in HIV care, overall, and by ART initiation status at each subsequent 12-month timepoint. These results are reported in Table 3, where we show that among those on ART by each censoring timepoint, the hazards of LTC were higher among those entering HIV care after the adoption of universal HIV treatment guidelines, than among those entering HIV care under prior guidelines. Among those with no record of ART initiation, the hazards of LTC after enrollment did not differ by timing of enrollment relative to guideline change.
- As the reviewer notes, retention on ART is not the same, and we therefore also examined patient retention at 12-month timepoints after ART initiation among the subset of patients with documentation of ART start. The results of this analysis are reported in what is now Table 4. Results show that the relative risks of retention at each 12-month timepoint were lower among patients initiating ART in the era of universal treatment, than among those initiating ART before guideline change.

**2. All before/after analyses have challenges with confounding by impacts that vary over time. This is also relevant in this study with its broad geographical scope and variable calendar time of the intervention. I believe that the authors should pay more attention to this in the Discussion. How do the authors assess the impact of external factors, including the C19 pandemic, the transition to INSTIs and possible other changes that may have happened in tandem with the TA policy, on the findings of their study?**

- Thank you for noting the potential impact of external factors. In our analyses of viral suppression among retained patients on ART with a viral load test, we did adjust for initial ART regimen type (see Methods, line 234), which we categorized as NNRT-based, Protease inhibitor-based, INSTI-based or other regimen, and as shown in Table 1 most patients in our study were in countries where Treat-All policies were adopted before 2016, meaning that the outcome ascertainment windows did not coincide with COVID-19. In our revisions, however, at the suggestion of another reviewer, we have included additional sensitivity analyses excluding countries with late adoption of universal HIV treatment guidelines where longer-term outcomes at 24 and 36 months could have partially coincided with service delivery disruptions brought about by the COVID-19 pandemic or with the rollout of INSTIs. Results from these sensitivity analyses (reported in Supplementary Tables 3 and 4) are consistent with our main analyses, which we have noted in our Results (lines 368-371) and Discussion (lines 401-403).

**3. The authors state that "our heterogeneous sample of patients from diverse settings across multiple regions and different years of Treat-All adoption" is a strength of the study. However, it seems difficult to compare the effects of the transition to TA in the USA to those in Africa several years later, with the vastly different health systems and populations. In my view, adjustment for income level of the country does not fully address this. For instance, readers may be concerned that the overall outcomes and conclusions could be less relevant for Africa due to being driven by results from North America and Asia. Could the authors elaborate on this?**

- Thank you for noting these important points. We agree that country income level does not fully address many facets of health systems and populations that may influence the outcomes under study, and we have noted this limitation in our Discussion (lines 438-441). Regarding whether results could be driven by North America and Asia, we have added the counts and proportions of patients from each IeDEA region in Table 1, and also added Footnotes to Table 1, listing the countries adopting universal HIV treatment guidelines in 2012-2015, 2016, and 2017-2018. We believe that these additional details, which show that patients from North America and Asia make up only 12.6% of the study population, suggests that study outcomes are not driven by results from those two regions.

**4. Enrolment of the post TA cohort was directly after the introduction of the change in policy. Could disruptions in health systems due to the change in policy have a temporary effect on the study outcomes? An example of this is supply challenges due to increased demand. If so, participants enrolled in the period immediately after the TA policy introduction may not be representative of individuals started on ART later.**

- A prior IeDEA analysis examining rapid ART iniitiation after the adoption of universal HIV treatment policies have shown that there were immediate and large absolute increases in rapid ART initiation in some settings, and in other settings where there was no immediate increase in rapid ART initiation, there were significant increases in the rate of rapid ART initiation in the months after guideline adoption [1]. Accordingly, it is possible that in many settings, an increased demand for and focus on treatment initiation contributed to resource constraints for other aspects of HIV care. We agree that this is an important limitation, and have added discussion of this point in our Discussion (lines 438-441).

**Reviewer #3:**

**This paper reports a comprehensive analysis of retrospective data on retention, viral suppression and other outcomes in a large multi-national cohort of patients enrolling in HIV care. The data come from the IdEA consortium and include information on nearly 67,000 patients from 109 clinics in 25 countries. The main objective of the analyses is to compare outcomes in patients enrolling before and after national guidelines changed to a Treat-All policy in each country.**

**This is a very well written paper. The rationale, methods and results are generally clearly presented and there is a good discussion of the policy implications of the findings. Strengths of the study include the availability of service delivery data from a very large sample of patients in a wide range of settings, with follow-up to 36 months after enrolment in HIV care. Previous studies have mostly been based on more restricted geographical settings and shorter follow-up periods, so this study makes an important contribution to the field. The findings of lower retention and little improvement in viral load monitoring in patients enrolling after the change to Treat-All have clear implications for policy and practice.**

**An important limitation is that there are no study sites in Southern Africa, where a large proportion of HIV patients live, although this reflects the coverage of the IdEA consortium, over which the authors have little control. Furthermore the study only includes data on patients enrolling in care during the first year after the guideline change. It usually takes time for guidelines to "bed in" and for health services to adapt to the new context, and especially the challenge of serving a larger number of patients on ART. It will be important to carry out further analyses once the new guideline has been in place for a longer time. These points are not discussed.**

- We appreciate Reviewer 3’s close review and thoughtful consideration of our manuscript. We agree that a limitation of the study is that it does not reflect all regions of the IeDEA consortium. As we have explained in our Methods section (lines 149-151), we included IeDEA regions that chose to participate in this study, and as a matter of practice, IeDEA regions and sites must opt in to participate in each proposed analysis. We have acknowledged and discussed this in our revision, as well as the fact that the lag in the availability of service delivery data meant that we could only examine outcomes among patients entering care and initiating treatment in the first year following adoption of universal treatment guidelines (lines 456-459). In our conclusion, we have also emphasized that our study was limited to patients entering care and initiating treatment in the first year after guideline change, and that ongoing monitoring of longer-term HIV care outcomes remains critical (lines 473-481).

**Apart from these observations, I only had a few comments which are listed below.**

**1. Some readers may be confused by the fact that WHO made the recommendations for Treat-All in 2015 and yet some sites included in the study changed to Treat-All as early as 2012. A brief comment on the reasons for this might be helpful.**

- Thank you for raising this important point. We have clarified in lines 118-121, that universal HIV treatment guideless were adopted in some countries prior to WHO’s 2015 recommendation and have replaced the term ‘Treat-All’ with “universal HIV treatment guidelines” throughout the manuscript to avoid potential confusion on this point.

**2. A key issue is that this is an observational analysis, and that the groups of patients enrolling before and after the guideline change may differ in ways that would affect the outcomes being measured, leading to selection bias. Table 1 shows that there are few differences in basic demographic and clinical variables, and the effect measures are adjusted for these variables. However, there may be differences in other unmeasured variables. For example, a Treat-All policy might encourage a wider range of patients to present for care, possibly including a higher proportion of those with intrinsically lower health-seeking behavior, and consequently lower retention and/or adherence to treatment. I note that hazard ratios are considerably attenuated after the limited adjustment that is possible, and might be further attenuated if it were possible to adjust for additional variables. This is mainly a point for discussion.**

- Thank you for noting these points. We agree that there may be differences between patients entering care before and during the era of universal treatment that we were unable to adjust for in this study, and we have acknowedged this in the Discussion section (lines 421-423, 435-438).

**3. Some of the analyses are stratified by whether or not patients had initiated ART before end of follow-up. But it is not clear how long they took to initiate ART, so how much of their follow-up period was while they were on ART. We are told that 87.6% of those enrolling in HIV care after the guideline change were on ART by 12 months, but some more information on the delay in initiating ART might be helpful.**

- Thank you for noting this point. We have added a row to Table 1, reporting the mean time to treatment initiation before and after guideline change among those initiating ART within 12 months of enrollment, and have referenced these data in our Results section (lines 280-284) and in the Discussion (lines 386-388).

**4. A smaller proportion of patients enrolling after the guideline change had CD4 counts at baseline, which is not surprising as ART initiation was no longer dependent on the CD4 count value. However, this lack of data compromises the ability to control for an important clinical variable. Again, this is mainly a point for discussion**.

- We agree that the decreased availability of enrollment CD4 measurements limits our ability to adjust for an important patient characteristic, and have acknowledged this in the Discussion (lines 435-438).

**5. Figs 3 and 4: The legend for these figures needs to explain that 95% CIs are shown and also define what is meant by "significant". Also, it would be possible to display the confidence intervals on the diagrams.**

- We have corrected the figure legends and noted the statistical tests, along with exact p-values. Additionally, we have reduced the size of the symbols used in Figures 3 and 4 to better display the confidence intervals. For some estimates, the confidence intervals are very narrow and remain difficult to discern visually.

**6. Table 3 does NOT show the "hazards" of LTC at each time-point. The first two columns show (I think) the cumulative incidence or risk of LTC by each time-point. It is in columns 3 and 4 I think that hazard ratios are shown. Could this be clarified please?**

- Thank you for noting this error. We have corrected the table heading.

**7. Table 4: A minor point, but with a covariate with several categories (e.g. age) it would be usual to show an LRT p-value for the overall evidence of any effect of that variable on the outcome, in addition to the effects and CIs for the individual categories.**

- Thank you for noting this. We have since removed the previous Table 4 altogether, at the request of Reviewer 1.

**8. Supplement Table 1: Please check the footnotes, which should define both HR and RR as these are both shown in the table.**

- We have added Footnotes to Supplemental Table 1 to define abbreviations used in the table. For Supplemental Table 2, whehere HR and RR were shown and defined, we have re-ordered the abbreviations in alphabetical order.

References

1.	Tymejczyk O, Brazier E, Yiannoutsos CT, Vinikoor M, van Lettow M, Nalugoda F, et al. Changes in rapid HIV treatment initiation after national "treat all" policy adoption in 6 sub-Saharan African countries: Regression discontinuity analysis. PLoS Med. 2019;16(6):e1002822-e.
