# Supplementary material for: Long-term HIV care outcomes under universal HIV treatment guidelines: A retrospective cohort study in 25 countries
Source: PLoS Med. 2024 Mar 18;21(3):e1004367. doi: 10.1371/journal.pmed.1004367 (PMC10962811; doi:10.1371/journal.pmed.1004367)
Supplement: Attachment — Submitted filename: Response to reviewers.docx [file pmed.1004367.s009.docx]

Richard Turner PhD

Louise Gaynor-Brook, MBBS PhD

Editors, PLOS Medicine

Dear Dr. Turner and Dr. Gaynor-Brook,

We would like to reiterate our thanks to you and the reviewers for considering our revised manuscript— “Long-term HIV care outcomes under universal HIV treatment guidelines: A retrospective cohort study in 25 countries” (PMEDICINE-D-23-02140R1)—and for the additional suggestions for improving this manuscript.

We have addressed the editors’ and reviewers’ comments point-by-point, noting line numbers in the clean manuscript file where these changes have been made. We have also uploaded a version of the manuscript with changes marked.

The information reporting under the Funding Statement and Competing Interests has also been corrected to address an error in one author’s initials and to ensure that our Funding Statement adheres to the journal’s guidance. In doing so, I observed that the PDF proof still contains outdated text under “Financial Disclosure.” I have been unable to find/access this Financial Disclosure field in the Editorial Manager platform and am not sure how to correct it to align with the information reported under Competing Interests. I would welcome advice about this.

We very much appreciate the close review and consideration of our manuscript, and we look forward to hearing back from you.

Sincerely,

Ellen Brazier, PhD

**Requests from Editors:**

**In the abstract and main text, we ask you to quote p values alongside 95% CI, where available.**

- Thank you for clarifying. We have added p-values to the abstract and main text, and also reported them for the adjusted estimates presented in tables 3 and 4, in addition to the 95% confidence intervals reported previously.

**At line 38 (abstract), we suggest "More than half ..." or similar.**

- We have made the suggested edit (line 38).

**At lines 85-86, we ask you to remove "25%" and "more than 30%", bearing in mind that these are relative increases. You may quote the relevant HR and CI if you wish.**

- We have removed the relative increases as suggested and added the relevant HRs and CIs (lines 84-88).

**We ask you to list the 25 countries, e.g., early in the Results section (main text).**

- We have listed the 25 countries included in the analysis (lines 270-273).

**At line 468 we suggest "While the adoption ... has ...".**

- We have made this correction (line 477).

**Noting information in the tables, please quote exact p values or "p<0.001" for the smaller values.**

- We have made these corrections to the tables.

**In the reference list, please use the journal name abbreviation "PLoS ONE".**

- We have made this correction to the respective references.

**Reference 3 may be missing page numbers or a PII: please add additional information as needed.**

- We have made this correction to Reference #3.

**To reference 18 and any other preprints, please add "[preprint]", or update the citation with the relevant published article.**

- We have made this correction.

**Comments from academic editor:**

**My comment regarding pregnant women and the provision of universal ART for this group from first antenatal visit which has been available for 2 decades (and from before Universal treatment guidelines for all became available) is not really covered by the sentence in the final paragraph of the discussion. This is not an issue of 'crowding out' but a concern as to how changes in this group contribute to the findings. This would be most likely in the African and South American context - but it would be nice to see whether there is any evidence of retention in care in this group was improved after treatment for all policy became the norm. I do not know if pregnancy status data were available in this dataset.**

- Thank you for this additional clarification and suggestion. Unfortunately, pregnancy status is not available for many of the cohorts included in this analysis and therefore was not part of our original analysis plan or the data made available for this study. As a result, it is not possible to examine whether retention among pregnant women improved after universal HIV treatment guidelines were adopted. We have acknowledged this limitation in our revision (lines 447-449), and also want to note that in our previous revision, we had noted the importance of monitoring long term HIV care outcomes, particularly among groups, such as pregnant and postpartum women, who are at increased risk of attrition and viral non-suppression (lines 488-491). We strongly agree that this is an important question worthy of study, and we will explore whether it is possible to undertake a future analysis focused on a subset of IeDEA countries and clinics for which pregnancy status is reliably reported in the data submitted to IeDEA.

**Comments from Reviewers:**

***** Reviewer #1:**

**I thank the authors for their responses to my comments on the previous version of this manuscript. I have a few follow-up questions and comments.**

- Thank you for the additional comments and suggestions for our manuscript.

**1. (This comment relates to the authors' response to my comment 3(b) on the previous version.) Please state in the Methods section that** **clustering of patients within clinics was accounted for when fitting the Cox proportional hazards models through the use of a robust sandwich estimator for the covariance matrix.**

- We appreciate this clarification and have incorporated the text recommended by the reviewer (lines 227-228).

**2. Were the assumptions of proportional hazards valid here?**

- Because of our large sample, we had difficulty running the Supremum test for assessing proportional hazards in SAS. However, as noted in Stensrud and Hernan’s recent commentary on testing proportional hazards assumptions [1], high p-values for this test may often mean that the test is underpowered. We therefore assumed that this test would likely yield a significant result because of our sample size and we relied on graphical methods—namely inspection of Kaplan-Meier curves for each group and all covariates, among all patients and stratified by ART status, along with plots of log-log Kaplan-Meier survival estimates against the log of follow-up time. These plots were reasonably parallel and did not show crossovers indicating violations of the proportional hazards assumption.

**3. In Table 1, rather than (or at least in addition to) including p-values to assess differences between the characteristics of patients enrolled before and after guideline adoption, I would recommend the calculation of standardised differences - these do not suffer from the issue of small difference + large sample size issue of p-values, and instead give some idea of the magnitude of the differences between groups.**

- We appreciate this suggestion and the fact that differences that are not clinically or programmatically substantive will be statistically significant in a large sample. However, in view of the specific guidance provided by the editor, we have focused on the reporting of p-values, while also adding in the additional statistics for continuous variables (standard deviations, medians and lower/upper quartiles) recommended by Reviewer 1 (see point 4 below). To our knowledge, standardized differences cannot be calculated for medians, and we were concerned that the table would be difficult to read and interpret if both p-values and standardized differences were reported for some, but not all characteristics presented in this table. In the results section of the paper, we emphasize those differences that are large enough in magnitude to be of potential programmatic significance.

**4. In Table 1, rather than 95% confidence intervals for the mean time in days to ART initiation, reporting means and standard deviations and medians and lower and upper quartiles would be more informative, since characteristics of the sample are being described.**

- We have modified Table 1 as suggested, reporting standard deviation for mean time to ART initiation instead of 95% confidence intervals, along with median and lower and upper quartiles. We have updated the text of the manuscript accordingly, reporting median time to ART initiation and lower and upper quartiles, instead of the mean and 95% confidence intervals (lines 289-290).

**5. The issue of the interpretation of p-values raised in point 4(c) of my review of the previous version of this manuscript does not appear to have been thoroughly addressed. For example, on page 9: lines 325-326 ("small, but significant"); line 332 ("significantly higher"); line 347 ("but differences did not reach statistical significance"). I recommend that these sentences be re-worded in terms of clinical significance of observed differences and the range of values supported by the data through the confidence intervals for estimates.**

- Thank you for flagging these statements. We have reworded each of these lines, as suggested, noting differences that were substantive or not. Where we felt that it was important to acknowledge that small differences were not statistically significant, we have done so (line 354).

**6. In Table 3, rather than noting which care outcomes had interaction terms with p-values below some arbitrary cut-off, please provide all interaction term p-values.**

- We have added the p-values for all interaction terms to Table 3.

***** Reviewer #2:**

**My comments and the extensive, very good comments from colleague reviewers, including those related to statistical issues, have been addressed well by the authors. Important limitations of the study remain but these have been acknowledged clearly in the Discussion. The conclusions are sufficiently supported by the data and are highly relevant for HIV care and treatment programs. In my view, the manuscript is ready for publication in PLoS Medicine**

- We appreciate the Reviewer’s consideration and feedback on our revised manuscript.

***** Reviewer #3:**

**I have carefully reviewed the revised version of the manuscript as well as the authors' responses to the reviews and editorial comments. I would like to commend the authors for the care with which they have revised the paper. I am very satisfied with the changes they have made in response to my comments. I only have the following final comments and suggestions but do not need to see the paper again:**

**1. I note that a suggestion was made to the authors to report more p-values for comparisons in the Abstract or Text. However, I do not agree with this suggestion. I take the same view as the authors, that while there is continuing debate on this issue, current best practice is to de-emphasize the reporting of p-values, especially in studies (like this one) where very large sample sizes mean that even the smallest observed differences are deemed "statistically significant". As the authors note, the Confidence Intervals provide much more useful information about the size and precision of the estimates of interest.**

- We appreciate the reviewer’s comments regarding the continuing debates surrounding p-values, and we have tried to balance views on this point with the requirements of the journal, which has emphasized the importance of including p-values where available.

**2. I commented previously that the term "hazard" is often used in the paper when what is meant is really "hazard ratio" - that is, an effect estimate (comparing different exposure groups). However, there are still instances (e.g. Line 344 and Table 3) where greater clarity would be achieved by using the term "hazard ratio".**

- Thank you for calling attention to these instances which we had overlooked in our revision. We have re-examined all places where ‘hazards’ or ‘hazard’ was used to confirm that the wording is correct.

**3. The lower proportion of patients with a viral load measure in the group enrolled after the guideline change is a surprising and concerning result that conflicts with the findings at 12m and 24m. I note that a much smaller number of patients in this group have data available at 36m, and suspect that they may be biased towards those enrolled at earlier time periods. If this is so, and if completeness of VL monitoring has been increasing over time, might this be an explanation for these contrasting results?**

- We agree that the difference in viral load monitoring at 36 months (aRR=0.86, 95% CI: 0.80, 0.92) conflicts with lack of difference we observed at 12 and 24 months. We also agree that there are considerably fewer patients with 36 months of follow-up time after ART initiation. Reasons why we might observe this particular difference at 36 months but not at 12 and 24 months include an increase in VL monitoring from 24-36 months in the pre-universal treatment group combined with a more pronounced decrease in VL monitoring from 24-36 months in the universal treatment group. Specifically, as shown in Table 2, we observed an increase from 70.3%-71.6% in the pre- universal treatment group, and a decrease from 73.4% to 63.7% in the universal treatment group. We investigated the decrease in the universal treatment group in the last round of revisions and noted in response to one of Reviewer 1’s earlier comments that we could rule out a potential biasing effect due to the COVID-19 due to later adoption of universal treatment polices. Specifically, “we did additional sensitivity analyses, excluding patients from countries with later adoption of universal treatment guidelines, and the results were consistent with our main analyses reported in Table 3 and Table 4.”
- In investigating this question further in response to this reviewer’s query, we excluded all patients initiating ART prior to 2013, when the World Health Organization recommended annual viral load monitoring—an exclusion that affects patients in high-income settings, such as the US and Canada where universal treatment guidelines were adopted ahead of the WHO ‘Treat All’ recommendation. Even when restricting the sample to this subset of patients who initiated ART in or after 2013, we observe that viral load monitoring is lower among patients initiating ART after adoption of universal treatment guidelines than among those initiating treatment in the pre-universal treatment era (60.0% vs. 68.3%, p<0.0001). As we noted in the manuscript, the observed difference in viral load monitoring is particularly surprising because the subset of patients enrolling in HIV care after guideline adoption with at least 36 months of follow-up time comprised more patients from high-income countries with greater—and earlier—capacity for viral load testing than other countries in our sample.
- We conclude that this is an important trend to continue monitoring as additional follow-up accrues in our cohort, including whether future clinical outcomes differ in patients with and without VL monitoring at 36 months.

References

1.	Stensrud MJ, Hernán MA. Why Test for Proportional Hazards? Jama. 2020;323(14):1401-2.
